# Rbm24a and Rbm24b Are Required for Normal Somitogenesis

**DOI:** 10.1371/journal.pone.0105460

**Published:** 2014-08-29

**Authors:** Samantha Maragh, Ronald A. Miller, Seneca L. Bessling, Guangliang Wang, Paul W. Hook, Andrew S. McCallion

**Affiliations:** 1 Biochemical Science Division, National Institute of Standards and Technology, Gaithersburg, Maryland, United States of America; 2 McKusick-Nathans Institute of Genetic Medicine, Johns Hopkins University School of Medicine, Baltimore, Maryland, United States of America; 3 Department of Molecular and Comparative Pathobiology, Johns Hopkins University School of Medicine, Baltimore, Maryland, United States of America; University of Sheffield, United Kingdom

## Abstract

We recently demonstrated that the gene encoding the RNA binding motif protein 24 (RBM24) is expressed during mouse cardiogenesis, and determined the developmental requirement for its zebrafish homologs Rbm24a and Rbm24b during cardiac development. We demonstrate here that both Rbm24a and Rbm24b are also required for normal somite and craniofacial development. Diminution of *rbm24a* or *rbm24b* gene products by morpholino knockdown resulted in significant disruption of somite formation. Detailed *in situ* hybridization-based analyses of a spectrum of somitogenesis-associated transcripts revealed reduced expression of the cyclic muscle pattering genes *dlc* and *dld* encoding Notch ligands, as well as their respective target genes *her7*, *her1*. By contrast expression of the Notch receptors *notch1a* and *notch3* appears unchanged. Some RBM-family members have been implicated in pre-mRNA processing. Analysis of affected Notch-pathway mRNAs in *rbm24a* and *rbm24b* morpholino-injected embryos revealed aberrant transcript fragments of *dlc* and *dld*, but not *her1* or *her7*, suggesting the reduction in transcription levels of Notch pathway components may result from aberrant processing of its ligands. These data imply a previously unknown requirement for Rbm24a and Rbm24b in somite and craniofacial development. Although we anticipate the influence of disrupting RBM24 homologs likely extends beyond the Notch pathway, our results suggest their perturbation may directly, or indirectly, compromise post-transcriptional processing, exemplified by imprecise processing of *dlc* and *dld*.

## Introduction

The *RBM* genes encode a diverse protein family defined by the shared presence of RNA-binding motifs (RBMs). The protein domain required for RBM classification is known by several names, including RNA recognition motif [1], RNA binding domain (RBD) or ribonucleoprotein [2] domain [3]. Little is known about the functions of the majority of RBM proteins. Recent reports on RBM3 [4], RBM4 [5], [6], RBM8A [7] and RBM38 [8], however, reveal potentially important developmental roles for these genes in such processes as craniofacial, pancreas, skeleton and muscle development. Thus far, dysfunctional *RBM* gene products have been causally implicated in four human developmental disorders. Null or hypomorphic *RBM8A* alleles cause thrombocytopenia with absent radii (TAR syndrome) [7]. X-linked syndrome talipes equinovarus, atrial septal defect, Robin sequence, persistence of the left superior vena cava (TARP syndrome) is caused by mutations in *RBM10* [9], whereas a heritable dilated cardiomyopathy in which the pre-mRNA of the cardiac splice variant of *TITIN* is incorrectly processed results from mutations in *RBM20* [10], [11]. Lastly, alopecia, progressive neurological defects and endocrinopathy (ANE syndrome) are caused by loss-of-function mutations in *RBM28* [12].

We recently identified *RBM24* as a gene of interest in early cardiac development, and evaluated the cardiac spatial and temporal expression of its homologs in mouse (*Rbm24*) and zebrafish (*rbm24a* and *rbm24b*). We demonstrated all assayed homologs to be expressed in the heart throughout cardiogenesis [13], [14]. We also showed that the zebrafish *rbm24a* and *rbm24b* genes were expressed in the earliest artery and vein, respectively, of the forming vasculature. We subsequently demonstrated that both Rbm24a and Rbm24b were required for normal cardiovascular development. In more recent work RBM24 has been reported to be necessary for sarcomere assembly and heart contractility [15].

In our initial description of the mouse *Rbm24*, we demonstrated that its expression was not limited to the heart but also included the somites [14]. Somites are developmental tissue blocks derived from the paraxial mesoderm. Structurally somites are paired tissue segments (one either side of the midline) along the trunk of the developing embryo, which formed from the posterior end of the embryo at regular intervals [16], [17]. In zebrafish somite segmentation occurs with a periodicity of 1 pair approximately every 30 minutes, from 10 hours post fertilization (hpf) to 24 hpf, with a total of 30 somite pairs formed. After somites segment, they begin patterning into differentiated cells and tissues. Somites give rise to skeletal muscle (myotome), cartilage of vertebrae (sclerotome), dermis (dermatome) and endothelial cells [18], [19], [20].

Somitogenesis begins after the completion of gastrulation when a niche containing multipotent mesodermal progenitor cells (MPCs), forms within the tailbud of the embryo. As an embryo continues to develop, MPCs being to specify and populate a zone called the pre-somitic mesoderm (PSM) just anterior to the tailbud. It is the PSM that provides cellular precursors that differentiate and organize into somite blocks. Maintenance of PSM cells and continuous somite segmentation with the appropriate periodicity and patterning is critical for normal skeletal muscle, cartilage, dermis and vascular development [18], [19]. Components of the WNT, FGF, SHH, BMP and NOTCH developmental pathways have been shown in model organisms to be required for normal somitogenesis [21], [22], [23], [24], [25], [26]. Zebrafish mutants for *gli2a*, *ntla*, *smad5*, *bmp7a*, *tbx6*, *tbx16*, *dlc*, *dld* or *notch1a* have dysregulated somitogenesis and aberrantly formed somites [27]. It is plausible that dysregulation of these critical developmental pathways underlies developmental disorders of as yet unknown etiology.

NOTCH-mediated signaling plays key roles in segmentation and somite patterning [28], [29], [30]. The NOTCH pathway acts via a complex multi-component path to achieve paracrine signaling. A simplified model of this pathway can be stated as cell surface ligands interacting with cell surface receptors on a neighboring cell to activate the expression of a target gene within that cell [28]. Specifically, ligands Dlc and Dld and target genes Her1 and Her7 have been shown in zebrafish to be critical components of somitogenesis and the segmentation clock, signaling within the PSM and immature segmenting somites [31], [32], [33], [34], [35]. The genes encoding these four proteins are commonly referred to as the somite clock genes. Cycling expression of these ligands and target genes persists through to the completion of somitogenesis and is partially maintained by negative feedback of target gene proteins on transcription of *dlc* and *dld* [31], [36]. Thus far Notch pathway dysregulation has been implicated as causal for two human disorders involving tissues of somite origin, Alagille Syndrome and spondylocostal dysostosis [37]. In Alagille Syndrome somitogenesis is believed to be impaired due to patients presenting with skeletal deformities and facial abnormalities with cardiac disease also prevalent. Several studies have linked loss of activity of the Notch ligand Jagged1 to the pathogenicity of Alagille Syndrome [38], [39], [40], [41]. Spondylocostal dysostosis represents a family of disorders all sharing the feature of short trunk dwarfism accompanied by vertebral segmentation defects along the length of the spinal column, with studies identifying mutations in Notch Ligand DLL3 as causal for the disorder [2], [37], [42].

In this study we investigate the functional requirement for RBM24 in somite development using the zebrafish model system. Our data support a functional requirement for the zebrafish Rbm24 homologs during somite development, and reveal perturbations in Notch pathway components as one possible contributor to phenotypes resulting from their titration *in vivo*. We provide evidence that disruption of zebrafish Rbm24 homologs can perturb pre-mRNA processing of the *dlc* and *dld* transcripts that encode Notch ligands in presomitic cell populations. Although we anticipate this impact is not specific to Notch pathway components it may, in part, explain some somitic deficits observed upon disruption of Rbm24 levels. These data present the zebrafish Rbm24 homologs as important players in the regulation of somite development, although as yet we do not understand the full spectrum of genes perturbed by knockdown of these *rbm24* genes. The potential impact of Rbm24 disruption on Notch pathway components also suggests it may be a reasonable candidate gene for human disorders in which Notch signaling is known (or hypothesized) to be disrupted.

## Results

### *rbm24a* and *rbm24b* are expressed in somites and presumptive skeletal muscle populations

We initially identified *Rbm24* in a screen for early cardiac genes and demonstrated it to be expressed at multiple time points during mouse cardiogenesis as well as in the somites (9.5 dpc) [14]. Our subsequent cardiovascular-focused studies in zebrafish determined a pivotal role for both zebrafish homologs (*rbm24a* and *rbm24b*) in the genesis of the cardiovascular system [13]. In addition to the reported cardiovascular defects, there was an apparent impairment to the development of other embryonic systems. Taking these data collectively we postulate that *rbm24a* and *rbm24b* may similarly contribute to the development of other systems in which they are expressed.

**Figure 1:**
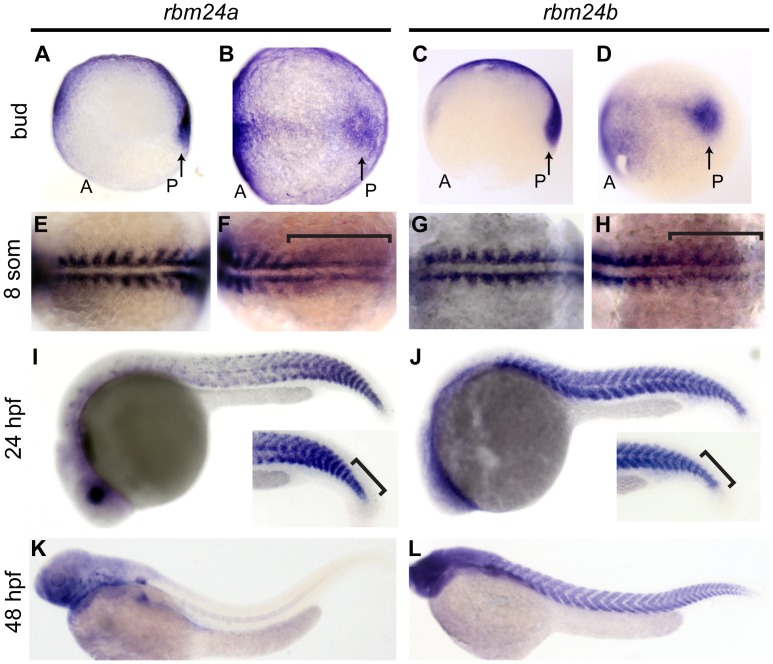
*rbm24a* and *rbm24b* are expressed throughout somitogenesis. *ISH* images of uninjected AB embryos using *rbm24a* or *rbm24b* riboprobes individually marking spatial and temporal RNA expression. Tailbud expression of bud stage embryos in lateral-slightly ventral facing (A, C) and dorsal (B, D) orientations. 8 somite embryos in dorsal facing orientations show all segmented somites (E, G) and PSM (F, H) marked by *rbm24a* and *rbm24b* riboprobes. Trunk images of embryos at 24 hpf show spatial expression of *rbm24a* in the somites concentrated at the posterior, while *rbm24b* is uniformly expressed in somites along the A-P axis (I–J). Inset of (I–J) highlights expression of *rbm24a* and *rbm24b* in the PSM. Embryos at 48 hpf show no somite *rbm24a* expression, while somite expression of *rbm24b* remains uniform thought the somites (K–L). som, somites; hpf, hours post fertilization; arrows, tailbud expression; A, anterior of embryo; P, posterior of embryo; bracket, PSM expression.

To address this question we assayed the embryonic expression of *rbm24a* and *rbm24b* in the embryo beginning at segmentation. We detected transcripts corresponding to both *rbm24a* and *rbm24b* in the tailbud at the bud stage and in somites during somitogenesis via antisense RNA *in situ* hybridization (*ISH*) (Figure 1). By 8 somites both *rbm24a and rbm24b* are expressed in segmented somites and in the PSM (Figure 1 E–H). Somitic and PSM expression of both *rbm24a* and *rbm24b* persists through 24 hpf at which time *rbm24a* transcripts are predominately localized in the most posterior somites (Figure 1 I,J). By 48 hpf, however, *rbm24a* expression is undetectable in the somites by *ISH* (Figure 1 K). By contrast, *rbm24b* is expressed uniformly throughout all somites at 24 hpf, and remains readily detected in the somites at 48 hpf (Figure 1 J,L).

In addition to their somitic expression, both *rbm24a* and *rbm24b* were also detected in developing craniofacial structures (Figure S1 A). The spatial expression pattern observed for each is consistent with multiple craniofacial muscle populations including those in the forming mandible, pharyngeal arches, otic vesicle and in optic muscles. Taken collectively these data are consistent with potential roles in development for both Rbm24a and Rbm24b beyond those previously described in cardiovascular development [13].

### Rbm24a and Rbm24b are required for normal somite and craniofacial development

**Figure 2:**
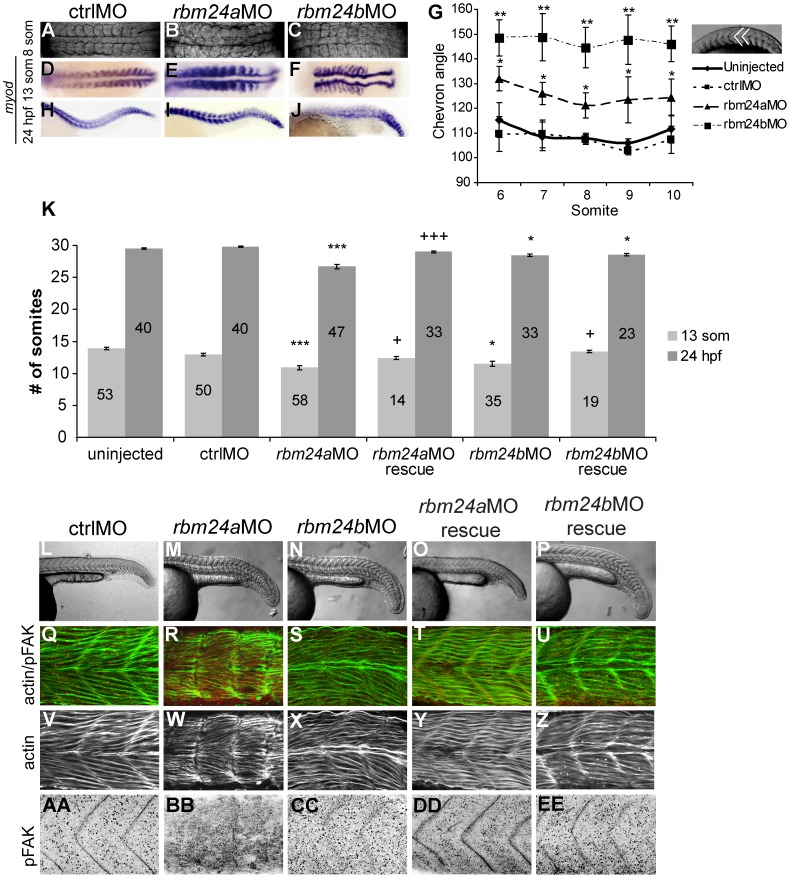
Rbm24a and Rbm24b are required for normal somitogenesis. Bright field 8 somite embryos are shown in dorsal orientation (A–C). Spatial expression of *myod* in somites of 13 somite embryos was detected using anti-sense riboprobes (D–F). Measurements of individual chevron angle measurements of somites 6–10 of 13 somite embryos for uninjected, ctrlMO injected *rbm24a*MO and *rbm24b*MO embryos, n = 3–5 embryos each (G). Statistically significant differences in somite angle as compared to ctrlMO is shown using an asterisk. The trunks of 24 hpf embryos are shown in the lateral orientation stained via *myod ISH* (H–J). Average somite counts of uninjected, ctrlMO injected *rbm24a*MO and *rbm24b*MO 13 somite and 24 hpf embryos are shown (K). Number of embryos counted shown on bars. Error bars are standard error of the mean. Significance determined at 95% confidence with significance compared to ctrlMO shown as * = p≤0.05; ** = p≤0.01; *** = p≤0.001 and significance between respective MO only and MO rescue conditions shown as + = p≤0.05; ++ = p≤0.01; +++ = p≤0.001. Trunks of 24 hpf uninjected, ctrlMO injected *rbm24a*MO, *rbm24b*MO, *rbm24a*MO embryos with *rbm24a* mRNA and *rbm24b*MO embryos with *rbm24b* mRNA embryos are shown in the lateral orientation imaged in bright field (L–P), double immunohistochemistry for phalloidin and anti-pFAK (Q–U), phalloidin alone (V–Z).

We evaluated the requirement for Rbm24a and Rbm24b during somite development, using morpholinos (MO) to elicit knock down of each protein product independently. We previously assayed the efficacy of both translation-blocking and splice-blocking MOs designed against *rbm24a* and *rbm24b* transcripts [13]. The translation-blocking MOs consistently displayed higher efficiency although both classes of MO resulted in the same phenotype [13]. We, therefore, used translation blocking antisense MO to evaluate the independent impacts of Rbm24a or Rbm24b knockdown on somite integrity and directly compared *rbm24a*MO and *rbm24b*MO injected embryos to embryos injected using a control non-targeting morpholino (ctrlMO). Reduction of either Rbm24a (*rbm24a*MO) or Rbm24b (*rbm24b*MO) disrupted normal somite patterning (Figure 2). At the 8 and 13 somite stages, *rbm24a*MO embryos frequently lacked distinct inter-somitic boundaries among multiple somites using the expression of the myogenic regulatory factor *myod* in somites as a marker. By contrast although somitic boundaries remained in *rbm24b*MO embryos their somites were laterally distended and demonstrated compression along the A–P axis (Figure 2 A–F). As further evidence of impaired somitogenesis in *rbm24a*MO and *rbm24b*MO embryos, the internal angle of somitic chevrons were measured in 13 somite stage embryos. The internal chevron angle was measured for somites 6–10 individually as representative for each embryo. We observed the internal angle of somitic chevrons to be significantly more obtuse among *rbm24a*MO (*p*≤0.037) and *rbm24b*MO (*p*≤0.0029) embryos when compared to ctrlMO embryos (n = 3–5 per treatment; Figure 2 G). There was no significant difference in chevron angle, however, between uninjected and ctrlMO injected embryos. By 24 hpf *ISH* for *myod* reveal the persistence of abnormal somite morphology in *rbm24a*MO (Figure 2 I). Although the effect of Rbm24a reduction on somite organization remained most marked in the posterior somites, consistent with localization of its expression, the structure of more rostral somites was also perturbed. Similarly, the effects of Rbm24b reduction were obvious at 24 hpf, with the integrity of truncal somites severely compromised (Figure 2 J).

We evaluated the somites present in *rbm24a*MO or *rbm24b*MO embryos at the 13 somite stage (∼15.5 hpf) and 24 hpf. Compared to ctrlMO embryos, there was a significant reduction in somites of *rbm24a*MO and *rbm24b*MO embryos at 13 somites (p≤0.0002, p≤0.031) and 24 hpf (p≤0.033, p≤2.2e-05) respectively. Abnormally formed somites were significantly more frequently observed in *rbm24a*MO and *rbm24b*MO embryos both at 13 somites and 24 hpf, compared to uninjected or ctrlMO injected embryos (Table 1). This suggests Rbm24a and Rbm24b may normally influence factors important for normal segmentation.

**Table 1:** Number of embryos with somite defects.

		13 somite			24 hpf			4 dpf		
Morpholino	Dosage	Embryos Studied	Somite Defects		Embryos Studied	Somite Defects		Embryos Studied	Craniofacial Defects	
uninjected	-	98	0 (0 %)		100	3 (3 %)		100	0 (0 %)	
ctrlMO	8 ng	89	8 (9 %)		107	3 (3 %)		49	1 (2 %)	
*rbm24a*MO	5 ng	91	65 (71 %)	***	91	74 (81 %)	***	91	79 (87 %)	***
*rbm24a* rescue	+800 pg RNA	44	29 (66 %)		119	45 (38 %)	+++	112	26 (23 %)	+++
*rbm24b*MO	8 ng	74	61 (82%)	***	72	59 (82 %)	***	72	62 (86 %)	***
*rbm24b* rescue	+50 pg RNA	61	42 (69 %)		71	44 (62 %)	+	70	18 (25 %)	+++

The number of embryos examined and number observed to have somite defects is listed for embryos at 13 somites and 24 hpf. Statistical significance and p-values were determined at a 95% confidence interval.

* = p<0.05 compared to ctrlMO.

** = p<0.01 compared to ctrlMO0.

*** = p<0.001 compared to ctrlMO.

+ = p<0.05 compared to respective MO.

++ = p<0.01 compared to respective MO.

+++ = p<0.001 compared to respective MO.

To determine if muscle structure and inter-somitic boundaries were altered at 24 hpf, embryos were stained with phalloidin to visualize actin fibers, and by immunofluorescence using an antibody against Phosphorylated FAK (Try397) (pFAK) to visualize inter-somitic boundaries [43]. Consistent with altered somite morphology, muscle structure in morphant embryos was severely disrupted and displayed wavy, disorganized, less tightly packed actin fibers than ctrlMO embryos. *rbm24a*MO embryos in particular showed a severe reduction of organized actin fibers (Figure 2 Q–S, V–X). We observed that morphant embryos had little/no detectable inter-somitic boundaries (Figure 2 AA–CC). These data indicate the patterning of somites, in addition to segmentation, is disrupted in *rbm24a*MO and *rbm24a*MO embryos.

In addition to these somite phenotypes, abnormalities in craniofacial development were also detected in *rbm24a*MO and *rbm24b*MO embryos consistent with their observed spatial and temporal expression (Figure S1 B). *rbm24a*MO and *rbm24b*MO embryos exhibiting somite defects by 24 hpf later developed abnormal craniofacial morphology, displaying a shortened mandible, reduction in the size of the otic vessicle, micropthalmia and microcephaly. Effects on the development of these structures, however, were again more pronounced in *rbm24a*MO embryos (Figure S1 B). Craniofacial muscle patterning at 72 hpf in mandibular, pharangeal arch, optic and fin bud muscle was reduced in both *rbm24a*MO and *rbm24b*MO embryos. Alcian blue cartilage staining at 96 hpf revealed a dramatic reduction of the craniofacial cartilage in *rbm24a*MO and *rbm24b*MO embryos yielding little to no signal corresponding to the ethmoid plate, palatoquadrate, hyomandibular and Meckel's cartilage (Figure S1 B). At 48 hpf and 96 hpf, concurrent with somite and craniofacial defects, we also observed our previously described cardiovascular defects in *rbm24a*MO and *rbm24b*MO embryos [13].

RNA rescue experiments were performed to confirm the specificity of MO phenotypes. Co-injection of *rbm24a*MO or *rbm24b*MO with their respective capped poly-A mRNA resulted in a significant decrease in the number of embryos exhibiting the observed somite phenotypes by 24 hpf (Table 1). By 13 somites segmentation delay is not detected in *rbm24a*MO rescue (p = 0.35) and *rbm24b*MO rescue (p = 0.30) where somite counts are not different from ctrlMO embryos (Figure 2 K). By 24 hpf *rbm24a*MO rescue and *rbm24b*MO rescue embryos showed actin filament organization similar to ctrlMO embryos and inter-somitic boundaries were detectable (Figure 2 T,U,Y,Z,DD,EE). Craniofacial phenotypes were also appropriately alleviated by 4 dpf at a higher frequency than somite rescue with at least 74% of *rbm24a*MO rescue and *rbm24b*MO rescue embryos showing like normal morphology (Figure S1 C).

These results suggest both Rbm24a and Rbm24b function early in somite/skeletal and craniofacial muscle patterning and are required for normal development. We focus here on understanding the requirement of both Rbm24a and Rbm24b in somite development because it is the earliest developing muscle population in which we observed both expression and developmental malformation.

### Notch-pathway transcripts are reduced in *rbm24a*MO and *rbm24b*MO embryos

Many pathways contribute to the orchestration of signaling in the PSM and subsequent somite/muscle formation and specification [21], [22], [44], [45]. Notch-mediated signaling is one such pathway that plays key roles in segmentation and somite patterning [28], [29], [30]. Specifically, ligands genes *dlc* and *dld* and target genes *her1* and *her7* are critical components of somitogenesis and the segmentation clock acting via cyclical cell-cell signaling within and the PSM and immature segmenting somites [31], [32], [33], [34], [35]. Dlc and Dld ligands function to activate transcription of *her1* and *her7* target genes in neighboring cells by signaling through Notch cell surface receptors [31], [33]. The normal functions of Her1 and Her7, include negative regulation of *dlc* and *dld* transcription that contributes to both perpetuation of the somite clock and maintenance of unsegmented cells within the PSM [22]. The expression of these genes cycle in both time and space from posterior PSM to anterior PSM and immature segmenting somites. The cycling expression of these ligands and target genes persists through to the completion of somitogenesis [30], [36], [46].

**Figure 3:**
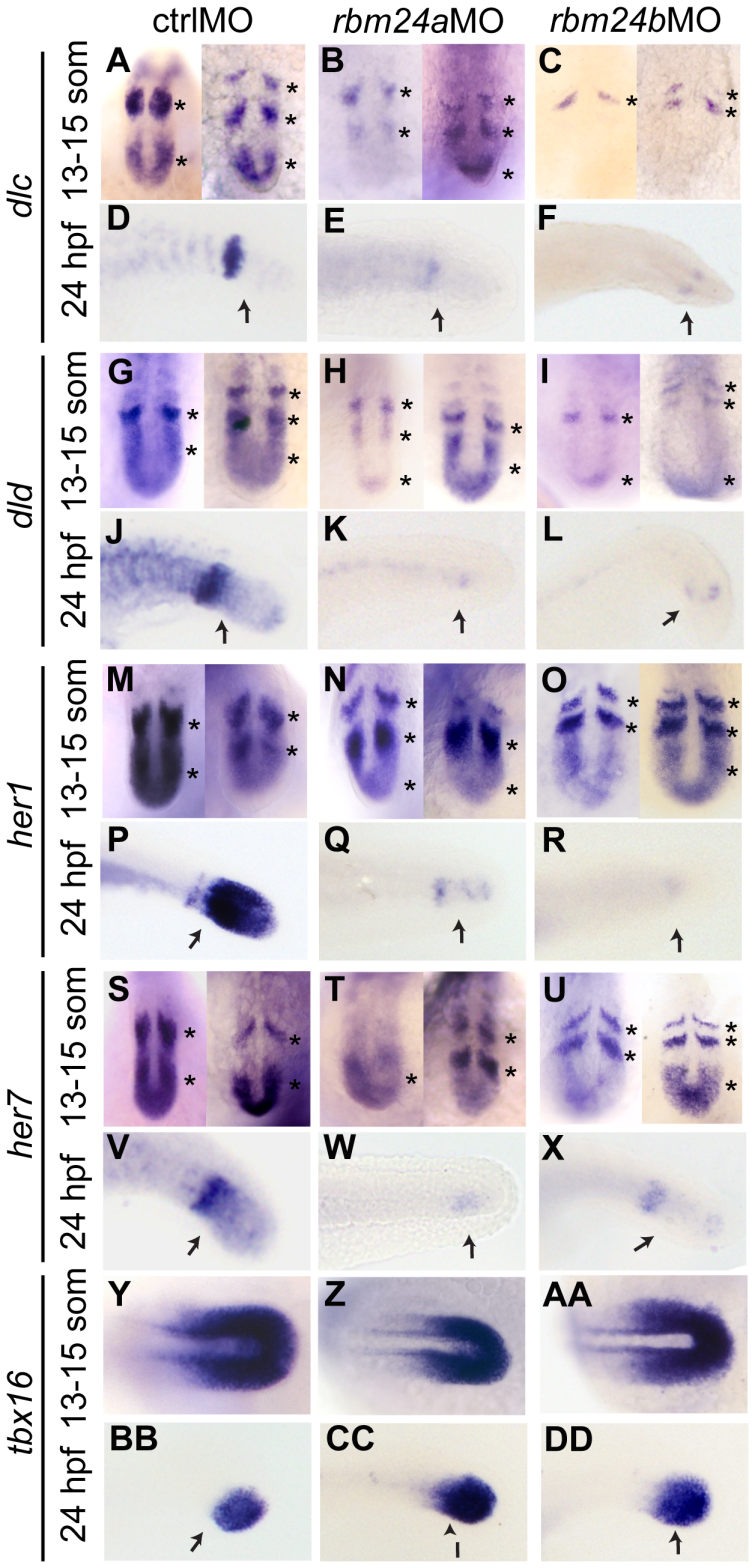
Notch-signaling pathway transcripts are depleted in somites of *rbm24a*MO and *rbm24b*MO embryos. *ISH* images of the PSM of dorsally oriented 13–15 somite and laterally oriented 24 hpf ctrlMO, *rbm24a*MO and *rbm24b*MO embryos. 13–15 somite embryos are shown in 2 different temporal stages of development. Spatial expression of *dlc* (A–F), *dld* (G–L), *her1* (M–R) and *her7* (S–X) and *tbx16* (Y–DD). Asterisks highlight cycling expression. n = 8–12 embryos stained per time-point with no more than 1 deviating from the displayed expression. arrow, regions of interest for PSM expression.

Given their demonstrable segmentation and inter-somitic boundary deficits, we investigated whether the Notch clock genes were perturbed in *rbm24* morphant embryos compared to ctrlMO embryos [43]. At the 13–15 somite stage two images are shown for each of the Notch pathway clock genes, with the first being expression during rostral bud extension, to demonstrate expression is cycling in space (Figure 3). Expression of *dlc* and *dld* ligand genes was diminished in the PSM in *rbm24a*MO embryos and almost ablated in *rbm24b*MO embryos at 13 somites Figure 3 A–C, G–I). Further both *dlc* and *dld* expression was nearly undetectable by 24 hpf (Figure 3 D–F, J–L). Expression of the target genes *her1* and *her7* in the PSM was mildly diminished in both *rbm24a*MO and *rbm24b*MO embryos at 13 somites (Figure 3 M–O, S–U), but then was almost undetectable by 24 hpf (Figure 3 P–R, V–X). Notwithstanding the observed reduction in *dlc*, *dld*, *her1* and *her7* expression, *tbx16* expression marking cells in the PSM was unchanged in *rbm24*MO embryos (Figure 3 Y–DD), indicating the reduced expression of Notch components does not a result from a lack of cells within the PSM. The observed reduction of *her1* and *her7* expression suggests it is unlikely that the observed reduced *dlc* and *dld* levels results from negative feedback on ligand transcription levels caused by increased *her1* or *her7* transcripts. In the PSM and immature segmenting somites, cell-cell signal transduction of Dlc and Dld ligands is mediated through cell surface receptors on neighboring cells [28], [47], and receptors Notch1a and Notch3 are known components of the somite clock [48], [49]. While we observed expression reduction of assayed ligands and target genes, we did not observe altered expression of *notch1a* or *notch3* transcripts (Figure S2).

We additionally evaluated whether observed *rbm24a*MO and *rbm24b*MO phenotypes were a consequence of gastrulation defects whose effects we were simply observing later in development [50]. We established if *rbm24a* and *rbm24b* are expressed prior to or during gastrulation. Expression of *rbm24a* and *rbm24b* was detected in 16-cell (cleavage), high (blastula), shield (gastrula) and 75% epiboly (gastrula) stage embryos (Figure S3). However, live imaging and *ISH* for embryos evaluated at 75% epiboly and bud stages showed no observable deleterious phenotypes suggesting gastrulation occurs comparatively normally in *rbm24* morphants (Figure S4). Later in development one consequence of gastrulation defects can be observed as an increase in notochord width [51]. However, we observed no difference between uninjected (38.37 µm+/−1.40), ctrlMO (37.72 µm+/−2.48), *rbm24a*MO (38.73 µm+/−1.12), and *rbm24b*MO (38.83 µm+/−1.23) embryos at the 13 somite stage (p>0.3). These observed results are consistent with the observed MO phenotypes arising post-gastrulation.

These studies indicate the transcripts for Notch ligands and their target genes, which are requisite for normal somitogenesis, are depleted as a consequence of knockdown of either *rbm24* homolog. These data implicate dysregulation of Notch signaling as a potential contributor to *rbm24a*MO and *rbm24b*MO phenotypes. Taking these findings together we further postulate disruption of Notch somite clock components is likely occurring at the level of ligand regulation.

### Aberrant *dlc* and *dld* transcripts are consistently detected in *rbm24a*MO and *rbm24b*MO embryos

Our findings thus far support a model in which reduction of either Rbm24a or Rbm24b results in both aberrant somitogenesis and concomitant reduction in the levels of the transcripts encoding Notch ligands, Dlc and Dld, along with those of their subsequent target genes, Her1 and Her7, in PSM and somite progenitors. We hypothesized that Notch signaling is being impacted at the ligand level. Recent reports suggest that the homolog for RBM24 in *C. elegans* (SUP-12) is a splicing factor active in muscle [52], [53], [54]. Additionally several other RBM proteins are known to function as post transcriptional regulators and splicing factors [10], [11], [55], [56], [57], [58], [59], [60], [61], [62]. While there have been no reports of RBM24 or other RBMs impacting the Notch pathway, based on the known role of RBMs in splicing we posited that a reduction of Rbm24a or Rbm24b would result in incorrect processing of pre-mRNA *dlc* and *dld* transcripts.

**Figure 4:**
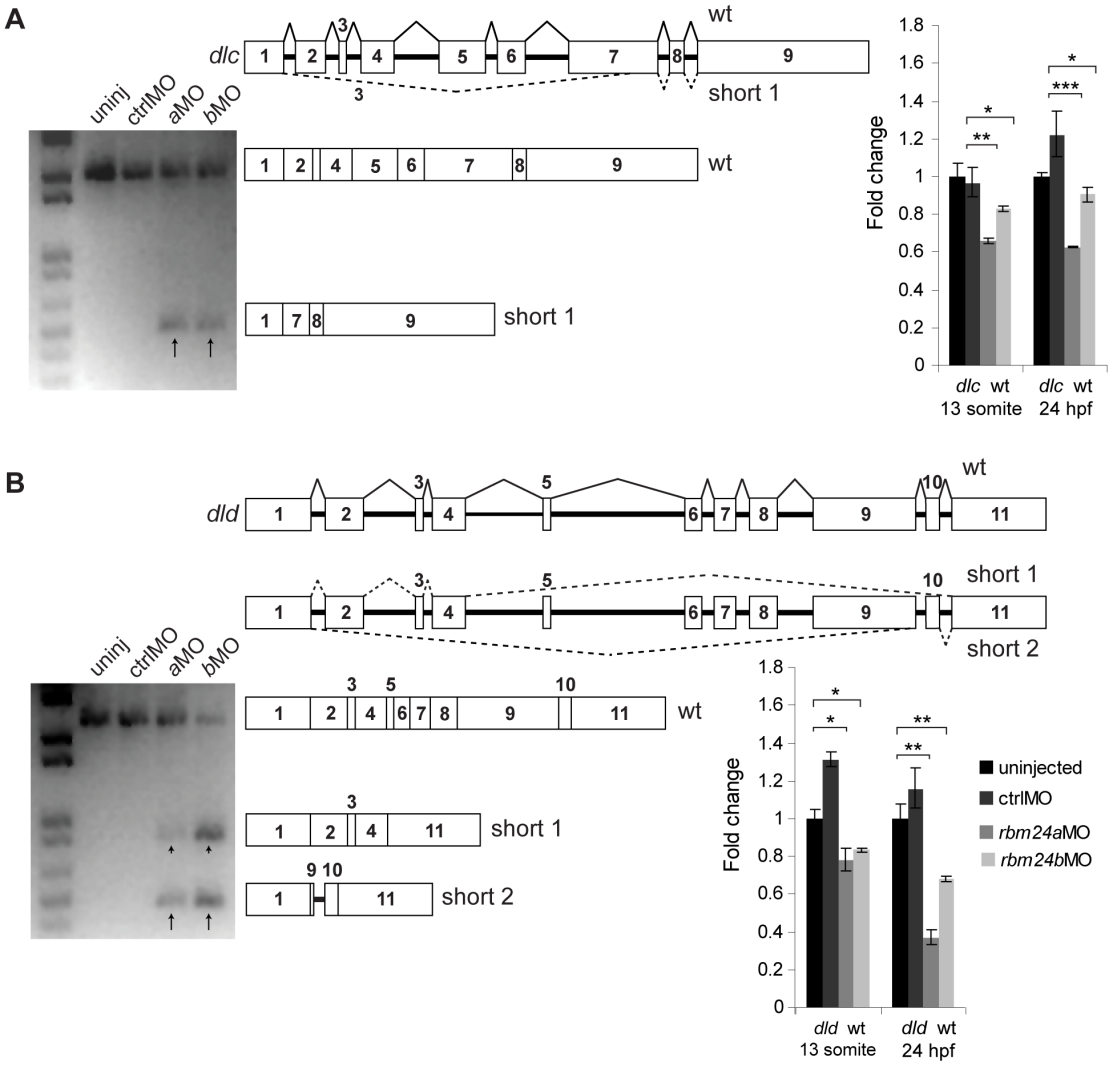
Aberrant *dlc* and *dld* splice forms are detectable in cDNA from *rbm24a*MO and *rbm24b*MO embryos. RT-PCR experiments to amplify *dlc* and *dld* mRNA transcripts using total cDNA generated from 13 somite uninjected, ctrlMO, *rbm24a*MO and *rbm24b*MO embryos (n = 50 embryos per condition). RT-PCR for *dlc* transcript yielded a fragment of correct length for all conditions and an additional short fragment for *rbm24a*MO and *rbm24b*MO cDNA (A). *dlc* pre-mRNA, *dlc* mRNA and *dlc* short 1 fragment sequences are depicted graphically to scale. RT-PCR for *dld* transcript yielded a fragment of correct length for all conditions and two additional short fragments for *rbm24a*MO and *rbm24b*MO cDNA (B). *dld* pre-mRNA, *dld* mRNA and *dld* short 1 and *dld* short 2 fragments are depicted graphically to scale. 13 somite and 24 hpf uninjected, ctrlMO, *rbm24a*MO and *rbm24b*MO embryos were used to generate cDNA used for qRT-PCR. All transcripts were assayed in triplicate per cDNA sample. Transcript levels were normalized to *elfalpha* transcript levels and fold change for each transcript is shown as compared to uninjected embryos. An asterisk denotes statistically significant fold changes compared to uninjected. * = p<0.05; ** = p<0.01; *** = p<0.001. black arrows, short *dlc* and *dld* fragments; boxes, exons; thick black lines, introns; thin black lines, wild type splicing, dashed lines, aberrant splicing.

To test this hypothesis we designed primers within the 5′ and 3′ UTRs of *dlc*, *dld, her1* and *her7* to amplify across the coding region of each transcript. Using random hexamer-generated cDNA from 13 somite and 24 hpf uninjected, ctrlMO, *rbm24a*MO and *rbm24b*MO embryos, we performed RT-PCR and detected fragments of the expected size for full length *dlc*, *dld, her1* and *her7* mRNA from all cDNA samples. However, in cDNA generated from *rbm24a*MO and *rbm24b*MO embryos we also detected additional fragments, shorter than the predicted sizes for *dlc* (*dlc* short 1) and *dld* (*dld* short 1 & *dld* short 2) transcripts (Figure 4). All *dlc* and *dld* short fragments were reproducibly detected in cDNA from both *rbm24a*MO and *rbm24b*MO embryos alone. No fragments in addition to wild type were detected for *her1* or *her7* under any treatment (data not shown). We subcloned and sequenced the short *dlc* and *dld* amplicons, revealing them to be abnormal transcripts variants of each respective pre-mRNA transcript. Sequence alignment of these three short fragments with the corresponding wild type mRNA showed aberrant processing occurred in 3/3 instances such that between five and seven exons were excluded bringing together upstream and downstream exons at positions in their sequence that also excluded their canonical splice donor and acceptor sites. Thus creating a unique junction joining two truncated exons (Table 2). Wild type *dlc* mRNA consists of exons 1–9 yielding 1,995 coding nucleotides (nt), while *dlc* short 1 consist of a truncated exon 1 (missing the last 11 nt) joined directly to a truncated exon 7 (missing the first 483 nt) with subsequent normal splicing to the inclusion of exons 8 and 9 for a coding length of 372 nt (Figure 4A, S5). Wild type *dld* mRNA consists of exons 1–11 yielding 2,154 coding nt, while *dld* short 1 consist exons 1–4 where exon 4 is truncated (missing the last 4 nt) joined directly to the 3′ UTR region of exon 11 such that the reading frame would include an additional 9 nt then a stop codon for a coding length of 612 nt; *dld* short 2 consists of a truncated exon 1 (missing last 8 nt) joined to a truncated exon 9 (missing the beginning 771 nt) followed by the retention of intron 9 (80 nt) and exon 10 with normal splicing and inclusion of exon 11 for a coding length of 256 nt (Figure 4B, S6).

**Table 2:** Short fragment junctions.

Exon	*dlc* wt mRNA coding nucleotides	*dlc* short 1 mRNA coding nucleotides	
1	254–304	254–293 (−11 nt)	
2	305–528	-	
3	539–596	-	
4	597–854	-	
5	855–1216	-	
6	1217–1433	-	
7	1434–2127	(−438 nt) 1917–2127	
8	2128–2242	2128–2242	
9	2243–2248	2243–2248	
Length	1,995 nt	372 nt	

Exon	*dld* wt mRNA coding nucleotides	*dld* short 1 mRNA coding nucleotides	*dld* short 2 mRNA coding nucleotides
1	461–508	461–508	461–498 (−10 nt)
2	509–805	509–805	-
3	806–866	806–866	-
4	867–1124	867–1120 (−4 nt)	-
5	1125–1185	-	-
6	1186–1317	-	-
7	1318–1486	-	-
8	1487–1703	-	-
9	1704–2502	-	(−773 nt) 2477–2502
10	2503–2608	-	(+80 nt intron 9 whole) 2503–2608
11	2609–2614	-	2609–2614
		(+12 nt 3′ UTR)	
Length	2154 nt	672 nt	256 nt

Exon boundaries are listed for each fragment detected and in Figure 4. Numbers in parenthesis notes truncated exons. Truncations on the 3′ end are listed to the right of exon lengths and truncations on the 5′ end are listed on the left of exon lengths.

Mapping of all potential splice donor and acceptor sequences that could yield the identified short fragments revealed no cryptic splice sites around the break points of the truncated exons [63], [64]. These aberrant fragments therefore appear to have been generated via an alternate mechanism as oppose to canonical splice site recognition. Further sequence analysis indicated none of the short fragments detected would be predicted to be functional. All short fragments lack motifs (delta-serrate domain or EGF-like domain repeats) required for the extracellular binding of Delta ligands to Notch receptors, and *dld* short 2 is also predicted to undergo nonsense-mediated decay. Using quantitative RT-PCR (qRT-PCR) on cDNA samples generated from 13 somite stage and 24 hpf embryos, wild type *dlc* and *dld* mRNA levels were significantly decreased in cDNA generated from *rbm24a*MO and *rbm24b*MO embryos but not ctrlMO embryos, compared to uninjected controls (Figure 4). We then assayed the relative abundance of the two aberrant *dld* mRNA splice forms, and found these fragments detectable by qRT-PCR in cDNA from *rbm24a*MO and *rbm24b*MO embryos. However, neither short variant RNA fragment was detectable in cDNA from uninjected or ctrlMO embryos (data not shown).

To investigate if other transcripts critical for normal somitogenesis might similarly produce aberrant transcripts in *rbm24a*MO or *rbm24b*MO embryos, RT-PCR was performed to amplify across the coding region of *fgf8a*, *gli2a*, *pax3a*, *smo* and *tbx6* transcripts. These transcripts were selected due to their similarity in coding length and exon number to *dlc* and *dld* transcripts as well mutants for these transcripts having somite morphology defects [18], [27]. Full coding length amplicons were observed for all transcripts with no unique fragments detected in cDNA generated from *rbm24a*MO or *rbm24b*MO embryos (Figure S7).

These data are consistent with a model in which a reduction of either *rbm24a* or *rbm24b* results not only in a significant reduction of wild type *dlc* and *dld* mRNA levels but also a molecular perturbation of the pre-mRNA transcripts of these genes by which there is production of aberrantly mRNA transcripts that are not readily detectable in uninjected or ctrlMO embryos. Our data suggest both *rbm24a* and *rbm24b* may participate in post-transcriptional processing of *dlc* and *dld*.

## Discussion

RBMs are a relatively poorly understood family of proteins, defined solely by the presence of RNA binding motifs [65]. To date, only a small subset of RBMs have been recognized to be functionally significant in humans and model organisms [4], [5], [6], [7], [8], [9], [10], [12]. Our recent work suggests that *RBM24*, encoding another family member, may similarly be developmentally critical [13].

We previously identified *Rbm24* as an early cardiac gene in mouse and interrogated the functional requirement of its zebrafish homologs Rbm24a and Rbm24b during cardiac development [13], [14]. We demonstrated both Rbm24a and Rbm24b were individually essential for normal cardiogenesis as well as vasculogenesis. Our initial cardiac-focused analyses in mouse demonstrated that in addition to the heart *Rbm24* was also expressed in somites. In this study we expanded upon our developmental analysis of *rbm24a* and *rbm24b*, focusing on the skeleto-muscular system. Here we demonstrate that *rbm24a* and *rbm24b* are prominently expressed in the forming somites, consistent with recent reports that RBM24 plays a role in myogenic differentiation [8], [66], [67]. Furthermore, we showed both genes to be expressed in developing craniofacial structures. Spatial expression localization of *rbm24a* and *rbm24b* was consistent with that of the skeletal muscle marker *myod*, suggesting *rbm24a* and *rbm24b* may be expressed in developing skeletal muscle tissue throughout the embryo. Subsequent depletion of either Rbm24a or Rbm24b resulted in somite and craniofacial malformation, while also recapitulating our previous report of cardiovascular defects.

Our findings suggest Rbm24a and Rbm24b are essential for skeletal muscle development in addition to their established roles in cardiac and vascular development. All of these tissues share origins in the mesoderm germ layer. It is, therefore, possible that mesoderm-derived populations are particularly sensitive to depletion of Rbm24a or Rbm24b. Our findings indicate this may be so for the multipotent cells of the PSM that pattern into somites. Although we cannot restrict the impact of Rbm24 reduction to only one pathway, we did observe a diminution of *dlc*, *dld*, *her1* and *her7* transcript levels normally localized in the PSM. These transcripts code for Notch-pathway components essential for normal somitogenesis.

Notch signaling is critical for the patterning of many embryonic tissues including the heart, hematopoeitic system and somites [29], [30], [33], [68], [69], [70]. During somite patterning the Dlc and Dld ligands function to activate Notch via of *her1* and *her7* target genes in neighboring cells in the PSM and immature segmenting somites [31], [33]. The normal functions of Her1 and Her7, include negative regulation of *dlc* and *dld* transcription which contributes to both perpetuation of the somite clock and maintenance of unsegmented cells within the PSM [22]. Mutants for *dlc*, *dld*, *her1* or *her7* exhibit de-synchronization of the somite clock, fused somites and loss of somite boundaries [22], [27], [36], [46]. The reported somite phenotypes of *dlc*, *dld*, *her1* and *her7* mutants appear more severe than, and overlap incompletely with, those we observed for *rbm24a*MO and *rbm24b*MO embryos in this study. While we did observed a loss of somite boundaries, we also observed compression of somites along the A-P axis and dorsal-ventral somite distortion, most notably in *rbm24b*MO embryos, which are not documented phenotypes of *dlc*, *dld*, *her1* or *her7* mutants. Taken together these observations indicate dysregulation of these components of the Notch pathway incompletely and accounts only partially for *rbm24a*MO and *rbm24b*MO somite phenotypes. Thus we anticipate that disruption of *rbm24a* or *rbm24b* also compromises other factors critical for normal somitogenesis.

In addition to the Notch pathway, the Wnt, FGF, Shh, and BMP developmental pathways are key participants in PSM regulation and somitogenesis and mutations in these pathways can also result in abnormal somite morphology [21], [22]. We postulate that Rbm24a and Rbm24b may similarly act upon targets within these pathways as a normal component of somitogenesis accounting for the differential phenotypes not expected to be due to Notch signaling pathway disruption, but also recognize the effects of Rbm24a and Rbm24b may not be restricted to these pathways.

Of those RBM members for whom functional data exists, several have been shown to be necessary for normal post-transcriptional processing. Indeed depletion of RBM4, RBM5, RBM11, RBM20 and RBM25 are known to be involved in aberrant post-transcriptional processing, and have been identified as splicing factors for known targets [10], [11], [55], [56], [58], [71]. Functional characterization of RBM9 has lead to the classification of this protein as a FOX family splicing factor and the re-naming of this protein as FOX2 [60], [61].

Our focus, on somitic expression of Notch-pathway components, lead us to identify aberrantly processed transcript fragments of the *dlc* and *dld* in *rbm24a*MO and *rbm24b*MO embryos. There has been one other report of an aberrantly processed *dlc* transcript, retaining the last intron, being unable to signal normally in somitogenesis [73]. The aberrant transcripts we observed resulted from the joining of distant and incomplete exons, skipping several intervening exons including those encoding domains required for Notch Delta ligand-receptor binding. This model is consistent with reports that the product of SUP-12, the RBM24 homolog in *C. elegans*, acts as a splicing factor in muscle that co-precipitates with the splicing complex [53], [54], [72]. However, none of the sequences flanking the observed transition points between truncated exons revealed any evidence of cryptic splice sites, suggesting that disruption of the fidelity of splice site recognition results from reduced levels of Rbm24a an Rbm24b. Alternatively the observed post-transcriptional processing defect may result (directly or indirectly) from disruption of nuclear homeostasis such that recognition of *dlc* and *dld* splice sites (among others) is rendered imprecise. We also recognize that depletion of Rbm24a and Rbm24b in the embryo may disrupt other developmentally critical pathways that contribute to these phenotypes.

Our data add to a growing body of literature highlighting the functional significance of RBM genes and the value of continuing to their continued study. Although translation blocking MO technology in zebrafish has been a powerful tool to begin functional characterization of Rbm24a and Rbm24b, we are cognizant that such MOs provide a surrogate for but not precise equivalent of an *in vivo* genetic model. Translation blocking MOs can inhibit both maternal and zygotic transcripts [74]. Our data indicates both *rbm24a* and *rbm24b* are expressed early in development both maternally and after the maternal-to-zygotic transition (Figure S3) [75], [76]. Hence, in future we are interested in characterizing the zygotic phenotype achieved through the generation of targeted mutant models.

We provide substantial evidence that Rbm24a and Rbm24b are required for normal somite biogenesis. Our data is consistent with a model in which disruption of Rbm24a and Rbm24b results (directly or indirectly) in abnormal post-transcriptional processing of Notch-ligand transcripts, *dlc* and *dld*, in turn providing a partial mechanistic explanation of the observed phenotypes. We acknowledge that depletion of Rbm24a and Rbm24b in the embryo may also impact other developmentally critical pathways that contribute to these phenotypes. It remains possible, of course, that the impact of *rbm24a/b* disruption on post-transcriptional processing may be indirect. Experiments are underway to determine the molecular mechanism through which these intriguing genes may act, and their relative importance.

## Methods

### Ethics Statement

All experiments were in accordance with ethical permits by Johns Hopkins Animal Care and Use Committee under protocol number FI10M369.

### Zebrafish Maintenance

Adult AB, zebrafish lines were maintained in system water according to standard methods (Westerfield, 1995). Embryos were obtained from natural mating of adult fish.

### Morpholino injection & mRNA rescue

*rbm24a*MO and *rbm24b*MO embryos were generated by injection of previously published *rbm24a* and *rbm24b* translation blocking antisense morpholinos into 1–2 cell stage embryos at 5 ng and 8 ng respectively [13]. The standard Gene Tools negative control morpholino oligo (CCTCTTACCTCAGTTACAATTTATA) was injected into 1–2 cell stage embryos at 8 ng to generate ctrlMO embryos. Double MO embryos were generated by co-injecting 2.5 ng *rbm24a*MO with 5 ng *rbm24b*MO in a single injection solution. mRNA rescue experiments were performed as previously described [13].

### Microscopy

Bright field and Fluorescence images were acquired on a Zeiss Luminar.V12 Stereoscope and a Nikon AZ100 dissecting microscope with white light. Zeiss Stereoscope images were analyzed with Zeiss AxioVision 4.8 software for embryo lateral length and somite angle measurements.

### Whole embryo *in situ* hybridization & Alcian Blue cartilage staining

Beginning at 24 hpf AB embryos were treated with 0.003% 1-phenyl-2-thiourea (PTU) to reduce pigmentation. Embryos were fixed in 4% paraformaldehyde in PBS overnight at 4°C. Antisense RNA *in situ* hybridization was performed on 75% epiboly, bud, 8 somite, 13 somite, 24 hpf, 48 hpf and 72 hpf zebrafish embryos with methods previously reported [13]. Riboprobe primer sequences are listed in table S1. Alcian blue cartilage staining was performed on 96 hpf embryos using published methodology [77].

### Fluorescent immunohistochemistry

Embryos at 24 hpf were fixed in 4% paraformaldehyde (PFA) overnight at 4 degrees and then processed as previously described [78], [79]. A mouse anti-Phospho-FAK (Tyr397) (1∶200, Invitrogen) was applied as the primary antibody, then a goat anti-mouse IgG conjugated Cy3 (Jackson ImmunoResearch Labs INC) was used as the secondary antibody at 1∶400 dilution. To visualize F-actin, phalloidin conjugated with Alexa Fluor 488 (1∶200, Life Technologies) was added with the secondary antibody. The dorsal somites were dissected from the embryo and mounted under a cover slip. Images were collected using a 20X objective on a Nikon A1-si Laser Scanning Confocal microscope.

### Splice variant RT-PCR and qRT-PCR

Total RNA was isolated from AB uninjected, ctrlMO, *rbm24a*MO and *rbm24b*MO whole zebrafish embryos at 13 somite and 24 hpf stages (n = 50 embryos per stage) using TRIzol Reagent. cDNA was generated from 2 µg RNA with random hexamers using the SuperScriptIII First-Strand Synthesis Kit (Invitrogen). RT-PCR was performed on embryo cDNA with primers designed to the 5′ and 3′ UTRs of *dlc*, *dld*, *her1* and *her7*. RT-PCR fragments were analyzed for size and Sanger sequencing was performed. qRT-PCR primers were designed to detect *elfalpha*, *dlc*, and *dld* wild-type transcripts as well as *dld* short 1 and *dld* short 2 transcripts. Primers for *dlc* and *dld* wild-type transcripts were designed in exons not present short *dlc* and *dld* transcripts. Primers to detect short *dld* fragments were designed across the unique splice junction present in these transcripts. qRT-PCR was performed in triplicate using Power SYBR Green (Applied Biosystems) on the Viia 7 Real-Time PCR System (Applied Biosystems). Fold amplification was determined via the delta-delta Ct method normalizing to endogenous control *elfalpha* and AB uninjected embryos. A students t-test was used to determine significance at p<0.05 *, p<0.01** and p<0.001***. RT-PCR and qRT-PCR primers are listed in table S1.

### Disclaimer

Certain commercial equipment, instruments, and reagents are identified in this paper to foster understanding. Such identification does not imply recommendation or endorsement by the National Institute of Standards and Technology, nor does it imply that the materials or equipment identified are necessarily the best available for the purpose.

## Supporting Information

Figure S1 **Rbm24a and Rbm24b are required for craniofacial development.** Bright field, *ISH*, and Alcian blue images of ctrlMO, *rbm24a*MO and *rbm24b*MO embryos. 72 hpf lateral oriented embryos show expression of *rbm24a* and *rbm24b* in presumptive optic muscles, pharyngeal arch muscles, mandibular muscles, and fin bud. *rbm24a* shows additional expression in the otic vesicle (A). The anterior region of 72 hpf and 96 hpf embryos are shown oriented laterally for visualization of eye and mandible phenotypes (B). Bright field and *ISH* images of *myod* expression in the anterior region of dorsally oriented 72 hpf ctrlMO, *rbm24a*MO and *rbm24b*MO embryos (B rows 1 and 2). *myod* expression is detected in the find bud, optic muscles, pharyngeal arch muscles and mandibular muscles of uninjected embryos. Normal *myod* expression is diminished in *rbm24a*MO and *rbm24b*MO embryos. Bright field and Alcian blue cartilage staining of ctrlMO, *rbm24a*MO and *rbm24b*MO embryos in lateral orientation at 96 hpf (B rows 2 and 3). Normal cartilage staining is observed in the fin buds, ethmoid plate, palatoquadrate, hyomandibular and Meckel's cartilage of uninjected embryos. Cartilage formation of these structures is ablated in *rbm24a*MO embryos and severely reduced in *rbm24b*MO embryos. Bright-field craniofacial images of RNA rescue *rbm24a*MO and *rbm24b*MO phenotypes at 96 hpf (C). *rbm24a*MO rescue, by co-injection of 5 ng*rbm24a*MO with 800 pg of capped poly-A *rbm24a* mRNA and *rbm24b*MO rescue, by co-injection of 8 ng*rbm24b*MO with 200 pg of capped poly-A *rbm24b* mRNA, show rescue of somite and craniofacial *rbm24a*MO and *rbm24b*MO phenotypes. fb, fin bud; om, optic muscles; ov, otic vesicle; ph, pharyngeal muscles; mm, mandibular muscles. white arrow, eye; black arrow, mandible; m, Meckel's cartilage; ep, ethmoid plate; pq, palatoquadrate; hm, hyomandibular cartilage.
(TIF)

Figure S2 ***notch1a*** **and** ***notch3*** **transcripts do not shown reduced tailbud expression in** ***rbm24a*** **MO and** ***rbm24b*** **MO embryos.** *ISH* of Notch pathway receptors transcripts *notch1a* (A–C) and *notch3* (D–F) in the somites of 24 hpf ctrlMO, *rbm24a*MO and *rbm24b*MO embryos.
(TIF)

Figure S3 ***rbm24a*** **and** ***rbm24b*** **are expressed before and after maternal-zygotic transition.** *ISH* of uninjected embryos with anti-sense and sense *rbm24a* and *rbm24b* riboprobes. Imaging of 16-cell (cleavage ∼1.5 hpf) (A–D), high (blastula ∼3.3 hpf) (E–H), shield (early gastrula ∼6 hpf) (I–L) and 75% epiboly (late gastrula ∼8 hpf) (M–P) show both *rbm24a* and *rbm24b* are expressed both before and after the maternal-to-zygotic transition.
(TIF)

Figure S4 ***rbm24a*** **MO and** ***rbm24b*** **MO embryos do not display gastrulation defects.** ctrlMO, *rbm24a*MO and *rbm24b*MO embryos during gastrulation. Bright field imaging of 75% epiboly embryos (A–C). Bright field imaging of bud stage (D–F). *ISH* of bud stage embryos with *tbx16* (G–I) and *ntla* (J–L) riboprobes.
(TIF)

Figure S5 ***dlc*** **RT-PCR fragment alignment to NM_ 130944.** *dlc* short 1 sequence is aligned to the refseq annotation for zebrafish *dlc*. Primers used for RT-PCR are highlighted in yellow. Primers used to make riboprobe are highlighted in green.
(PDF)

Figure S6 ***dld*** **RT-PCR fragment alignment to NM_ 130955.** *dld* short 1 and *dld* short 2 sequences are aligned to the refseq annotation for zebrafish *dld*. Primers used for RT-PCR are highlighted in yellow. Primers used to make riboprobe are highlighted in green.
(PDF)

Figure S7 **RT-PCR** ***fgf8a, gli2a, pax3a, smo, tbx6.*** RT-PCR experiments to amplify the coding region of *fgf8a, gli2a, pax3a, smo, tbx6* mRNA transcripts using total cDNA generated from 13 somite uninjected, ctrlMO, *rbm24a*MO and *rbm24b*MO embryos (n = 50 embryos per condition). RT-PCR for all transcripts yielded full coding length amplicons with no additional unique fragments detected in r*bm24a*MO or *rbm24b*MO embryos.
(TIF)

Table S1 **Primer sequences.** Primers used to generate antisense DIG labeled riboprobes, conduct splice variant RT-PCR and conduct qRT-PCR are listed.
(PDF)
