# Supplementary figures and images for: Rbm24a and Rbm24b Are Required for Normal Somitogenesis
Source: PLoS One. 2014 Aug 29;9(8):e105460. doi: 10.1371/journal.pone.0105460 (PMC4149414; doi:10.1371/journal.pone.0105460)

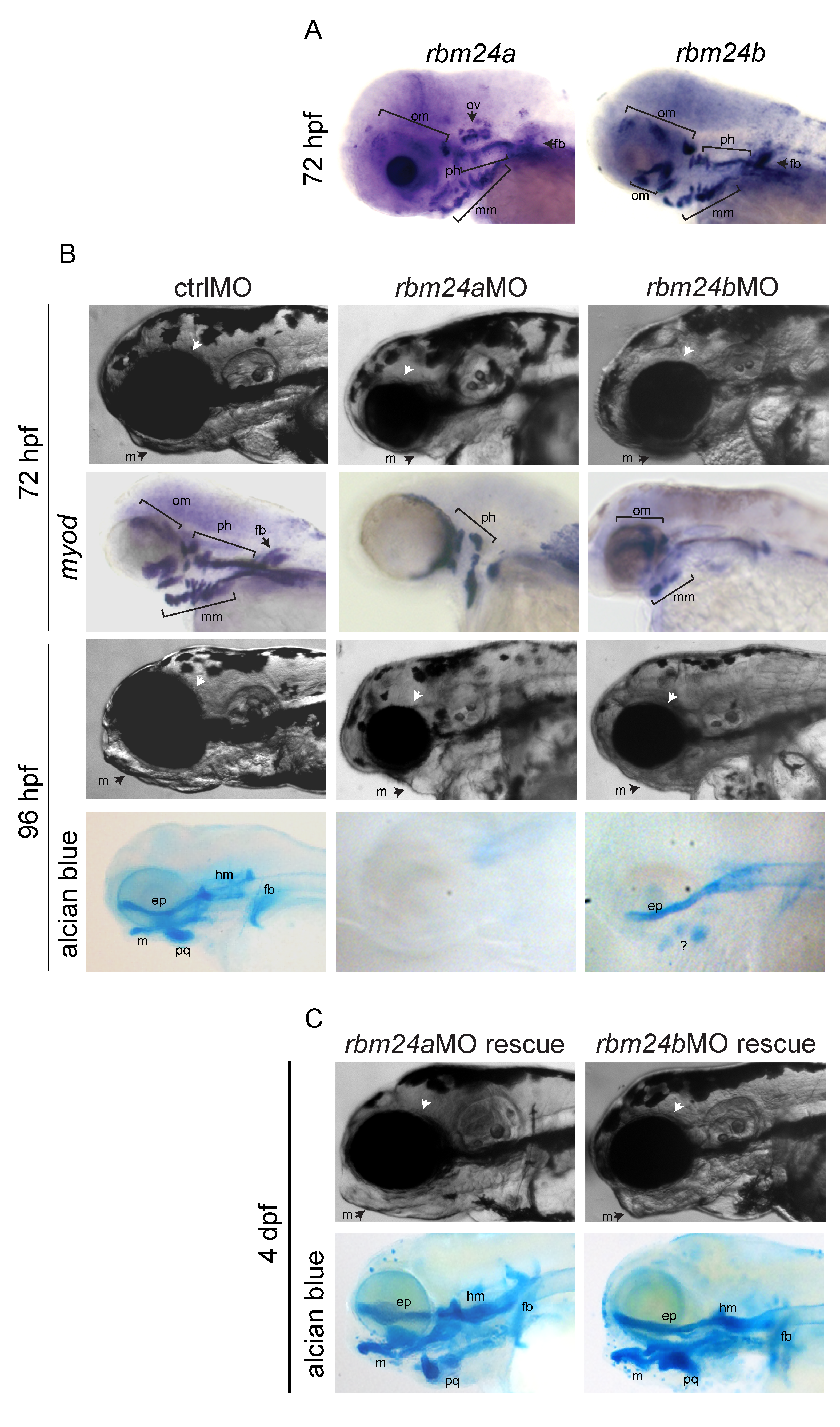

Supplement: Figure S1 — Rbm24a and Rbm24b are required for craniofacial development. Bright field, ISH, and Alcian blue images of ctrlMO, rbm24aMO and rbm24bMO embryos. 72 hpf lateral oriented embryos show expression of rbm24a and rbm24b in presumptive optic muscles, pharyngeal arch muscles, mandibular muscles, and fin bud. rbm24a shows additional expression in the otic vesicle (A). The anterior region of 72 hpf and 96 hpf embryos are shown oriented laterally for visualization of eye and mandible phenotypes (B). Bright field and ISH images of myod expression in the anterior region of dorsally oriented 72 hpf ctrlMO, rbm24aMO and rbm24bMO embryos (B rows 1 and 2). myod expression is detected in the find bud, optic muscles, pharyngeal arch muscles and mandibular muscles of uninjected embryos. Normal myod expression is diminished in rbm24aMO and rbm24bMO embryos. Bright field and Alcian blue cartilage staining of ctrlMO, rbm24aMO and rbm24bMO embryos in lateral orientation at 96 hpf (B rows 2 and 3). Normal cartilage staining is observed in the fin buds, ethmoid plate, palatoquadrate, hyomandibular and Meckel's cartilage of uninjected embryos. Cartilage formation of these structures is ablated in rbm24aMO embryos and severely reduced in rbm24bMO embryos. Bright-field craniofacial images of RNA rescue rbm24aMO and rbm24bMO phenotypes at 96 hpf (C). rbm24aMO rescue, by co-injection of 5 ngrbm24aMO with 800 pg of capped poly-A rbm24a mRNA and rbm24bMO rescue, by co-injection of 8 ngrbm24bMO with 200 pg of capped poly-A rbm24b mRNA, show rescue of somite and craniofacial rbm24aMO and rbm24bMO phenotypes. fb, fin bud; om, optic muscles; ov, otic vesicle; ph, pharyngeal muscles; mm, mandibular muscles. white arrow, eye; black arrow, mandible; m, Meckel's cartilage; ep, ethmoid plate; pq, palatoquadrate; hm, hyomandibular cartilage. (TIF) [file pone.0105460.s001.tif]

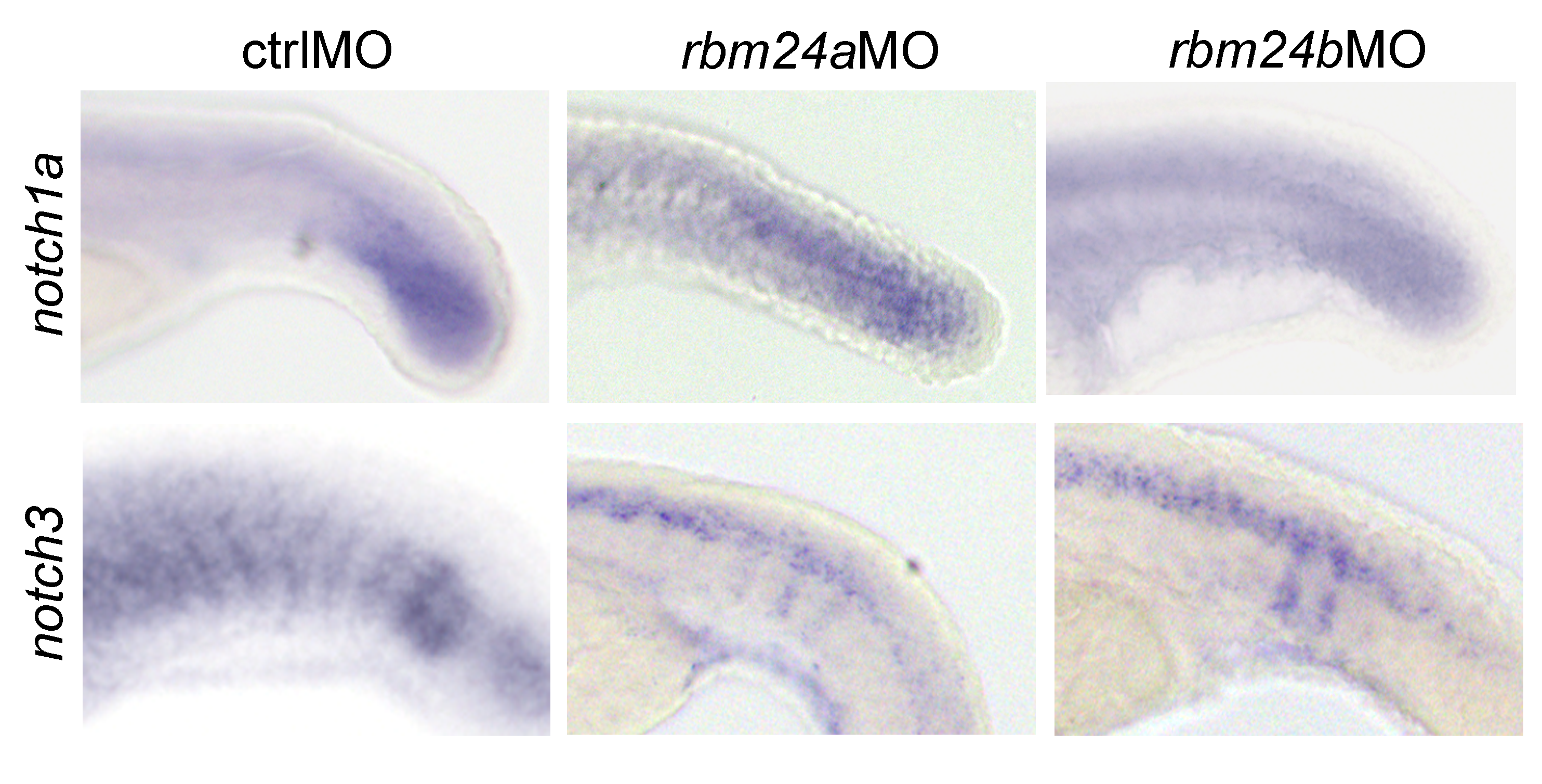

Supplement: Figure S2 — notch1a and notch3 transcripts do not shown reduced tailbud expression in rbm24a MO and rbm24b MO embryos. ISH of Notch pathway receptors transcripts notch1a (A–C) and notch3 (D–F) in the somites of 24 hpf ctrlMO, rbm24aMO and rbm24bMO embryos. (TIF) [file pone.0105460.s002.tif]

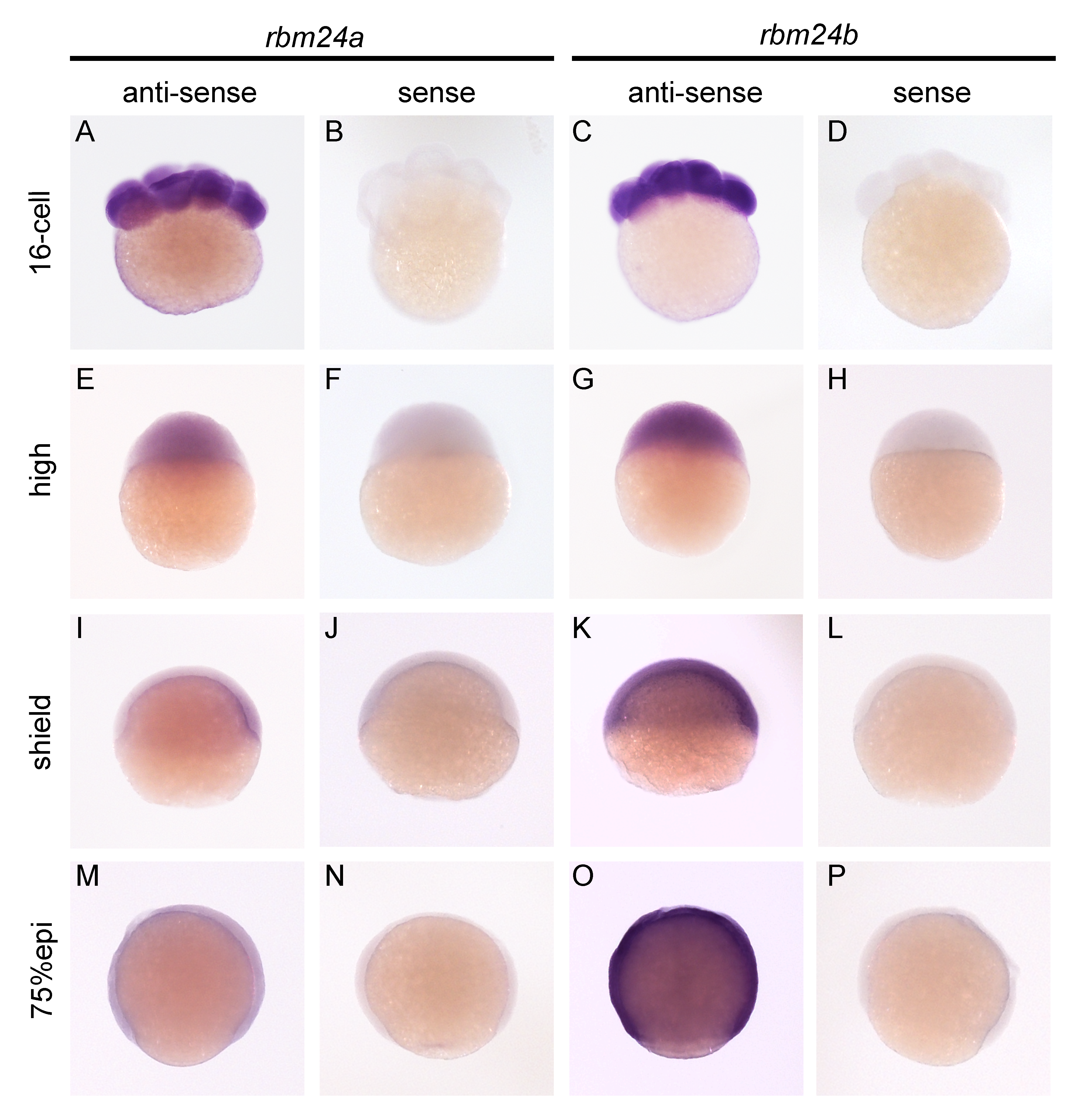

Supplement: Figure S3 — rbm24a and rbm24b are expressed before and after maternal-zygotic transition. ISH of uninjected embryos with anti-sense and sense rbm24a and rbm24b riboprobes. Imaging of 16-cell (cleavage ∼1.5 hpf) (A–D), high (blastula ∼3.3 hpf) (E–H), shield (early gastrula ∼6 hpf) (I–L) and 75% epiboly (late gastrula ∼8 hpf) (M–P) show both rbm24a and rbm24b are expressed both before and after the maternal-to-zygotic transition. (TIF) [file pone.0105460.s003.tif]

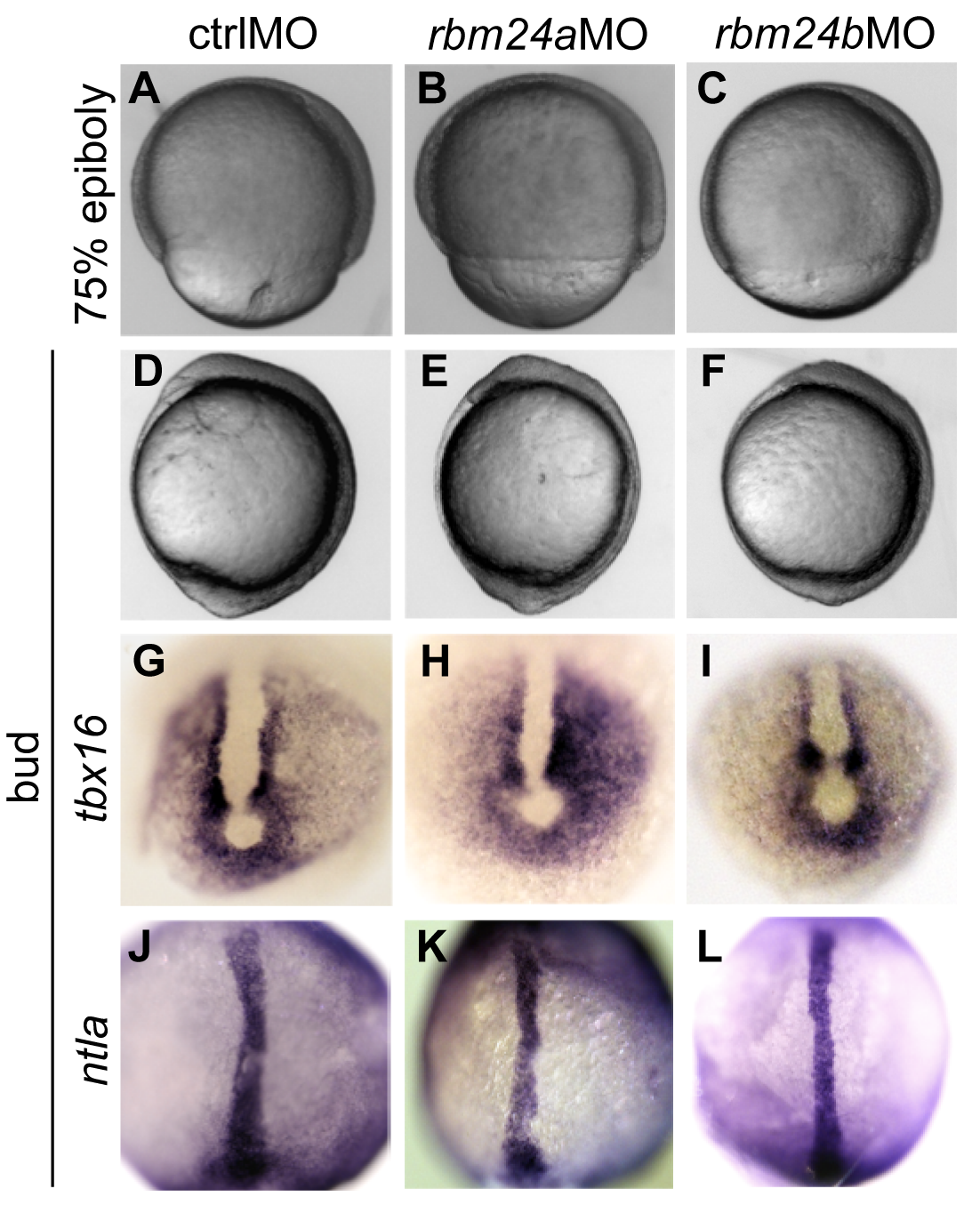

Supplement: Figure S4 — rbm24a MO and rbm24b MO embryos do not display gastrulation defects. ctrlMO, rbm24aMO and rbm24bMO embryos during gastrulation. Bright field imaging of 75% epiboly embryos (A–C). Bright field imaging of bud stage (D–F). ISH of bud stage embryos with tbx16 (G–I) and ntla (J–L) riboprobes. (TIF) [file pone.0105460.s004.tif]

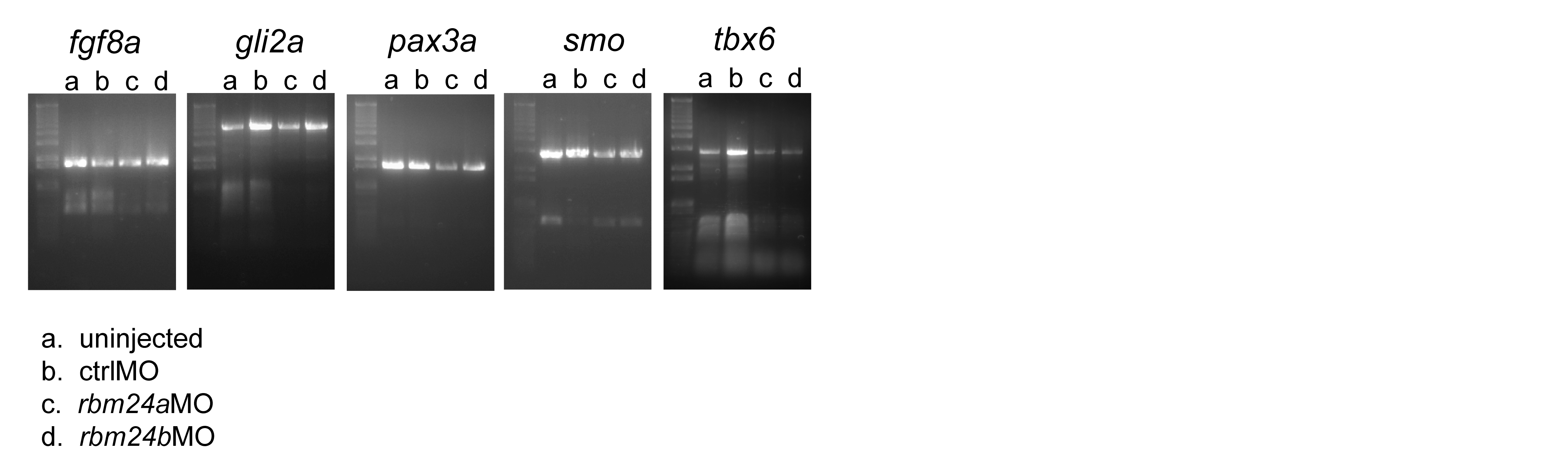

Supplement: Figure S7 — RT-PCR fgf8a, gli2a, pax3a, smo, tbx6. RT-PCR experiments to amplify the coding region of fgf8a, gli2a, pax3a, smo, tbx6 mRNA transcripts using total cDNA generated from 13 somite uninjected, ctrlMO, rbm24aMO and rbm24bMO embryos (n = 50 embryos per condition). RT-PCR for all transcripts yielded full coding length amplicons with no additional unique fragments detected in rbm24aMO or rbm24bMO embryos. (TIF) [file pone.0105460.s007.tif]
