# Supplementary material for: Rbm24a and Rbm24b Are Required for Normal Somitogenesis
Source: PLoS One. 2014 Aug 29;9(8):e105460. doi: 10.1371/journal.pone.0105460 (PMC4149414; doi:10.1371/journal.pone.0105460)
Supplement: Figure S5 — dlc RT-PCR fragment alignment to NM_ 130944. dlc short 1 sequence is aligned to the refseq annotation for zebrafish dlc. Primers used for RT-PCR are highlighted in yellow. Primers used to make riboprobe are highlighted in green. (PDF) [file pone.0105460.s005.pdf]

dlc mRNA short fragment alignment

```
dlc wt   1      GGGTTTTGAAGAAACGTAACTGAAGGGTCCAAAGTCCCCTGGAGCTAGAAATCTGTTTTTATTTGCTTTG  70
dlc wt   71     TAGGCTCCAAACACATGCGACATTGAGATACATTTTTATAAGAAATGTTCATTCGTTTAACTTTTTAACT  140
dlc wt   141    TTACGCATTTTTACAACTACTTTTTCCCTTTAAATTGTGGATAATACTTCAAACATTCATATCGGACTAC  210
short 1                                                                    TATCGGACTAC

dlc wt   211    TCTCACAGTCTGCTATCGTTCAGTAGCAGACAAGAAGGCAAAGATGGCTCGTGTTTTATTAACGTGCTTT  280
short 1         TCTCACAGTCTGCTATCGTTCAGTAGCAGACAAGAAGGCAAAGATGGCTCGTGTTTTATTAACGTGCTTT

dlc wt   281    TTTATTTTGATATCATCGCATCTGGGGAAATCATCCGGTGTGTTTGAGTTGAAAGTTCTGTCTTTCACAA  350
short 1         TTTATTTTGATAT---------------------------------------------------------

dlc wt   351    GCACGAGCAGTGTGTGTAAAGGGTCCAGCGACTGCCAGATCTTTTTCCGTGTTTGCCTGAAGCACTCGCA  420
short 1         ----------------------------------------------------------------------

dlc wt   421    AGCACTCATATTACCTGAGCCGCCGTGCACCTACGGCACCGGAATGTCAGAAATACTGAGCGCGGACTCC  490
short 1         ----------------------------------------------------------------------

dlc wt   491    ATCTCCAGCAGTGCGTATATAAGCGTGCCTTTTAATTTCAAGTGGCCGGGAATCGTCTCTTTGATAATCG  560
short 1         ----------------------------------------------------------------------

dlc wt   561    AAACCTGGAACGCAGAAACCTCTGACCAGTCAACAGAGAATAACAACAACATGATAAGCCGTTTGGCCAC  630
short 1         ----------------------------------------------------------------------

dlc wt   631    CAAAAGAAGACTCGCTATCAGTGAGGACTGGTCTCAGGACGTGCATCTCGGTCGACAAAGCCAACTGCGC  700
short 1         ----------------------------------------------------------------------

dlc wt   701    TTTTCTTATCGTGTCGTGTGCGATGAATTCTACCACGGCGAGGAATGCTCGGATTTCTGCCGCCCACGGA  770
short 1         ----------------------------------------------------------------------

dlc wt   771    ATGATACCTTCGGCCACTTCAACTGTGACGCCGCTGGCAACAGAATTTGCCTTCCTGGATGGAAAGGCGA  840
short 1         ----------------------------------------------------------------------

dlc wt   841    TTATTGCACCGAACCCATCTGCTTGTCTGGCTGTAGTGAGGAGAACGGTTATTGTGAGGCCCCCGGTGAG  910
short 1         ----------------------------------------------------------------------

dlc wt   911    TGCAAGTGCCGGATTGGATGGGAAGGCCCCCTCTGTGATGAGTGCACGCGGCACCCGGGGTGCTTGCATG  980
short 1         ----------------------------------------------------------------------

dlc wt   981    GCACCTGCAACCAGCCTTTTCAGTGCACTTGCAAAGAAGGTTGGGGCGGTCTGTTTTGCAATGAGGATCT  1050
short 1         ----------------------------------------------------------------------

dlc wt   1051   GAACTTTTGCACTAATCACAAGCCCTGTAGAAATGACGCCACGTGTACCAACACCGGCCAGGGCAGCTAC  1120
short 1         ----------------------------------------------------------------------

dlc wt   1121   ACCTGCATTTGCAAGCCTGGCTTCAGTGGCAAAAACTGTGAGATCGAAACCAATGAGTGTGACAGCAACC  1190
short 1         ----------------------------------------------------------------------

dlc wt   1191   CCTGCAAGAATGGAGGCAGTTGCAATGACCAGGAGAATGATTACACTTGTACATGCCCGCAAGGCTTCTA  1260
short 1         ----------------------------------------------------------------------

dlc wt   1261   TGGCAAGAACTGCGAGGTCAGCGCCATGACCTGTGCCGATGGACCCTGCTTCAATGGTGGAACCTGCATG  1330
short 1         ----------------------------------------------------------------------

dlc wt   1331   GAGAAGGGATCCGGTAGCTATTCCTGCCGCTGCCCTCCTGGATACATGGGCTCCAACTGTGAGAAGAAAA  1400
short 1         ----------------------------------------------------------------------

dlc wt   1401   TCGACCGGTGCAGCAGTGACCCCTGTGCTAACGGTGGCCAGTGTCTCGATTTGGGCAACAAAGCGACGTG  1470
short 1         ----------------------------------------------------------------------

dlc wt   1471   CCGTTGCCGGCCCGGGTTCACAGGCTCACGTTGTGAAACAAACATTGACGACTGCTCAAGCAACCCCTGT  1540
short 1         ----------------------------------------------------------------------
```

```
dlc wt   1541  CAAAATGCAGGCACCTGCGTGGATGGTATCAACGGGTACACCTGCACATGCACGCTTGGTTTCTCAGGCA  1610
short 1        ----------------------------------------------------------------------

dlc wt   1611  AGGACTGTAGGGTTCGCTCTGACGCCTGCAGTTTCATGCCCTGCCAGAACGGAGGAACCTGCTACACTCA  1680
short 1        ----------------------------------------------------------------------

dlc wt   1681  CTTCTCTGGGCCTGTCTGCCAGTGCCCGGCAGGCTTCATGGGCACACAGTGCGAGTACAAACAGAAGCCC  1750
short 1        ----------------------------------------------------------------------

dlc wt   1751  ACGCCTGTGAACAGCCCTGCTCTTCCAGCAGCCTTAATAGTCTCATTTACTCTAGGCCTCATTACTCTGA  1820
short 1        ----------------------------------------------------------------------

dlc wt   1821  CCTTAGTGATCTGTGCTGCCATTGTGGTCCTGCGACAGATGCGTCAGAACCACAAAGCCAGCTCAACCAC  1890
short 1        ----------------------------------------------------------------------

dlc wt   1891  AGTTCGAAACAACCTGGATTCTGTCAATAATCGCATTTCTTTGAGCCCAACCTCACCTTTAGGTAGAGAG  1960
short 1        ----------------------------AATCGCATTTCTTTGAGCCCAACCTCACCTTTAGGTAGAGAG

dlc wt   1961  AAGGAGGCCTTCCTTATTCCTGGTGGCCCATTTAAGGTGTCCAATAAAGATATGGCGCTCAGATCCACCT  2030
short 1        AAGGAGGCCTTCCTTATTCCTGGTGGCCCATTTAAGGTGTCCAATAAAGATATGGCGCTCAGATCCACCT

dlc wt   2031  CTGTAGACACACATTCCAGTGACAAATCAAACTATAAGCAGAAGATGGTGGACTACAATCTGAGCATTGA  2100
short 1        CTGTAGACACACATTCCAGTGACAAATCAAACTATAAGCAGAAGATGGTGGACTACAATCTGAGCATTGA

dlc wt   2101  TGAAAAGCACACAAACAACAAACTAGAGAAAAACTCTGAATCAACATTGCTGGTTCCACCTTTAAACTAT  2170
short 1        TGAAAAGCACACAAACAACAAACTAGAGAAAAACTCTGAATCAACATTGCTGGTTCCACCTTTAAACTAT

dlc wt   2171  CCAAAAGAGGGAGTGTATCATCCTGTGTACATCATTCCCGAACACATAGAACAACGTGTGTTTGCTACTG  2240
short 1        CCAAAAGAGGGAGTGTATCATCCTGTGTACATCATTCCCGAACACATAGAACAACGTGTGTTTGCTACTG

dlc wt   2241  AGGTATAGCAGAATTCTTCAATCTGGAGCACCTCAAACACCAGTGGGCCTGACCACAAAAATGTTTACAG  2310
short 1        AGGTATAGCAGAATTCTTCAATCTGGAGCACCTCAAACACCAGTGGGCCTGACCACA

dlc wt   2311  TCTCTAAGGACAAAAGGAAGACGTGAAGAGAGTATATATCACATGCATTATATTATTTATTTATTCATGC  2380
dlc wt   2381  TGCTCACTAAATGGACTACTTCTTGATATGGATAATGAATTTGTGAAGACAAGCAAGAGACTTGAAGACC  2450
dlc wt   2451  CGAGGAACAACTTGCATTATTTGCACTATTTCCTTTCCCTTTTTTAATGACCCAAACAGAATTTTTATAT  2520
dlc wt   2521  ATTTGTCATCATATTTAAATTGTACATAGACACAAGAACTCTCTGGAAGAGCTGAAGAATCAAGGATTTG  2590
dlc wt   2591  ATAATCACAGAAGCCTTGAAAAGGAAGAAAATAGGCATCCTGAAGTTCATCTAACTCTGTGAATCACATG  2660
dlc wt   2661  AAGCCCAAACAATCAGCCTTATGGGTGGAGGAGGTGCAACTGAAGGATCTGGAACTGTGACTGTGGAGTA  2730
dlc wt   2731  TTTGCCTTTTATTTTTATTTTTTCGATATGGTTCGAAATGCTATTTATAAATGGAACTGTGATGAGAAGC  2800
dlc wt   2801  AATCAAACTGAACAAAACAAGTTGTTCTGGCTGTGAACAATGTGAACAACTGTCTTGGAAATGTACAGTT  2870
dlc wt   2871  TTATGCGGCAGTGATTTTACTTAGTGCCTACAATGGATCTGACTATCAGTGACATTTTCTCAAACCAAAC  2940
dlc wt   2941  CCAATCTCAATTAAGTGCCTCATTCTTTAATGTGCTACAGCTCAGTTACAAAGGTACTCCTTAGAAACAC  3010
dlc wt   3011  TGAAAAGAGCCTTTTTTTAAACACTATAGGAGTATGCTTTTCGACAGTAGATGTTTGGGTTGATTTGAGT  3080
dlc wt   3081  ACAGTTGTTTAAACAGCGATGGATGATGGATGCTGTTGTGTACAGCGGCCATCCTGTCAACATTCACTGG  3150
dlc wt   3151  TGGTAACTATTTCCGACATTCCCAAGGCTTGAAAGGACCCTTGGTCCAAACTGATAGCCTCAACATTGTC  3220
dlc wt   3221  AAGATTTCCTGGGTTTGTAATCCTCTGCAATCAGAGTGTTTTTCAAAATTCACAACAACATAGATTGTTC  3290
dlc wt   3291  TATGTCTTTTCCTTTCCTATTTTATTTTTTATTTTTTAGTATGTGGCATTTGTTCCGTCATTTTCATTTT  3360
dlc wt   3361  TTAAAACCCTTTAAACCCAAAGAGGTGGAACCTTTAATATTTTAAACCGTTTTCCTTAATTTACCAATGC  3430
dlc wt   3431  CATAAGGGCCAAATTTTTGGATAATCCCCTATTTAATTGGGCCGTAAAAATTTTGGATTTATGAGAGGAT  3500
dlc wt   3501  GAATGGATATAAGGTTAAGGATTTTGCAAATATATATATATATTTTTTCAAATAAATAAACCCCCGGAAT  3570
dlc wt   3571  AATTTAAAAAAAAAAAAAAAAAAAAAAAAAAAAAAAAAAAA                               3610
```
