# Supplementary material for: Rbm24a and Rbm24b Are Required for Normal Somitogenesis
Source: PLoS One. 2014 Aug 29;9(8):e105460. doi: 10.1371/journal.pone.0105460 (PMC4149414; doi:10.1371/journal.pone.0105460)
Supplement: Figure S6 — dld RT-PCR fragment alignment to NM_ 130955. dld short 1 and dld short 2 sequences are aligned to the refseq annotation for zebrafish dld. Primers used for RT-PCR are highlighted in yellow. Primers used to make riboprobe are highlighted in green. (PDF) [file pone.0105460.s006.pdf]

dld mRNA short fragment alignments

```
dld wt    1      AGTCAGTCTCTCACATGGAAACAAGAGTACGGGATGAAAGTTTGGATCAGTAGTTTGGATCATCTCCACA  70
dld wt    71     TGAACTTTTGAAACGGAAAATAAGCGCATAAATAACCCTGTGCTTGGGGTTTTGTCATTTTTCCGGCTTC  140
dld wt    141    CTAAACTACCCGAAAGTGGACTGCTAACTTTTTTTGACAACTTGGAGTACTCGACTTGTGAGGGATTTCC  210
dld wt    211    CAATCGTCTCCCTTTTGCGCATCGGGGTTTCCTCCTTCTACATTCGTATTTGGGATTTAATTGCTGATTT  280
dld wt    281    ATTTTCCAAGGGGGGACTTTGATGGGCTTGCATGAGCAGATAAACTGAACACAACAGAGCATCAACCCGA  350
dld wt    351    GCTGCCTCCCACTGAGCGGAGAACGACGAGTCTCAATGATTTATTGAGACACGGTCTCACACAATTGCCT  420
short 1                                                                               TGCCT
short 2                                                                               TGCCT

dld wt    421    GGCCCGTGGAAGTTTCACAACAGTTCAGATCCTCTCCGCCATGGGACGACTAATGATAGCTGTTTTGCTT  490
short 1          GGCCCGTGGAAGTTTCACAACAGTTCAGATCCTCTCCGCCATGGGACGACTAATGATAGCTGTTTTGCTT
short 2          GGCCCGTGGAAGTTTCACAACAGTTCAGATCCTCTCCGCCATGGGACGACTAATGATAGCTGTTTTGCTT

dld wt    491    TGTGTCATGATAAGCCAGGGGTTTTGTTCAGGGGTTTTTGAGCTAAAGTTGCAAGAGTTTCTGAACAAGA  560
short 1          TGTGTCATGATAAGCCAGGGGTTTTGTTCAGGGGTTTTTGAGCTAAAGTTGCAAGAGTTTCTGAACAAGA
short 2          TGTGTCA---------------------------------------------------------------

dld wt    561    AAGGAGTGACAGGCAACGCAAACTGCTGCAAGGGATCCGCGGCAGAGGGTCTTCAGTGTGAATGCAAAAC  630
short 1          AAGGAGTGACAGGCAACGCAAACTGCTGCAAGGGATCCGCGGCAGAGGGTCTTCAGTGTGAATGCAAAAC
short 2          ----------------------------------------------------------------------

dld wt    631    TTTTTTTAGGATTTGCCTGAAACATTACCAAGCCAACGTATCTCCAGATCCTCCGTGCACCTACGGTGGC  700
short 1          TTTTTTTAGGATTTGCCTGAAACATTACCAAGCCAACGTATCTCCAGATCCTCCGTGCACCTACGGTGGC
short 2          ----------------------------------------------------------------------

dld wt    701    GCAGTTACCCCGGTGCTCGGATCAAACTCCTTCCAAGTTCCCGAAAGCTTCCCTGACAGCTCGTTCACCA  770
short 1          GCAGTTACCCCGGTGCTCGGATCAAACTCCTTCCAAGTTCCCGAAAGCTTCCCTGACAGCTCGTTCACCA
short 2          ----------------------------------------------------------------------

dld wt    771    ACCCCATTCCTTTCGCGTTTGGGTTCACATGGCCAGGAACATTTTCGCTGATTATTGAAGCGCTGCACAC  840
short 1          ACCCCATTCCTTTCGCGTTTGGGTTCACATGGCCAGGAACATTTTCGCTGATTATTGAAGCGCTGCACAC
short 2          ----------------------------------------------------------------------

dld wt    841    CGACTCCACTGATGACCTGTCTACAGAAAACCCAGACCGTCTGATCAGTCGCATGACCACCCAGAGGCAT  910
short 1          CGACTCCACTGATGACCTGTCTACAGAAAACCCAGACCGTCTGATCAGTCGCATGACCACCCAGAGGCAT
short 2          ----------------------------------------------------------------------

dld wt    911    CTAACAGTAGGCGAGGAATGGTCCCAAGATCTACAGGTTGGTGGGAGGACAGAGCTGAAGTACTCATACA  980
short 1          CTAACAGTAGGCGAGGAATGGTCCCAAGATCTACAGGTTGGTGGGAGGACAGAGCTGAAGTACTCATACA
short 2          ----------------------------------------------------------------------

dld wt    981    GATTCGTTTGTGATGAGCATTACTACGGCGAGGGCTGCTCGGTCTTCTGCCGTCCGCGCGATGATACTTT  1050
short 1          GATTCGTTTGTGATGAGCATTACTACGGCGAGGGCTGCTCGGTCTTCTGCCGTCCGCGCGATGATACTTT
short 2          ----------------------------------------------------------------------

dld wt    1051   CGGCCACTTCACCTGCGGAGAGCGCGGAGAAATTATCTGCAACTCCGGATGGAAAGGACAGTACTGCACA  1120
short 1          CGGCCACTTCACCTGCGGAGAGCGCGGAGAAATTATCTGCAACTCCGGATGGAAAGGACAGTACTGCACA
short 2          ----------------------------------------------------------------------

dld wt    1121   GAACCAATCTGTCTTCCGGGGTGTGATGAAGACCATGGCTTTTGCGACAAACCCGGTGAATGCAAATGCA  1190
short 1          ----------------------------------------------------------------------
short 2          ----------------------------------------------------------------------

dld wt    1191   GAGTAGGATTTAGTGGAAAGTACTGTGACGACTGCATTCGCTACCCAGGCTGCTTGCATGGCACCTGCCA  1260
short 1          ----------------------------------------------------------------------
short 2          ----------------------------------------------------------------------

dld wt    1261   ACAGCCCTGGCAATGCAACTGCCAAGAGGGTTGGGGAGGTCTCTTCTGTAACCAAGATCTCAATTACTGC  1330
short 1          ----------------------------------------------------------------------
short 2          ----------------------------------------------------------------------
```

```
dld wt    1331  ACACATCACAAACCGTGCCAGAATGGAGCCACTTGCACCAACACAGGCCAGGGAAGCTACACCTGCTCAT  1400
short 1         ----------------------------------------------------------------------
short 2         ----------------------------------------------------------------------

dld wt    1401  GCAGACCTGGCTTCACCGGGGACAGCTGTGAGATTGAGGTCAACGAATGCTCCGGCAGCCCGTGCAGAAA  1470
short 1         ----------------------------------------------------------------------
short 2         ----------------------------------------------------------------------

dld wt    1471  TGGAGGAAGTTGCACTGATCTTGAAAACACCTACAGCTGCACTTGTCCTCCTGGTTTCTACGGAAGAAAC  1540
short 1         ----------------------------------------------------------------------
short 2         ----------------------------------------------------------------------

dlc wt    1541  TGCGAGCTGAGTGCCATGACTTGTGCCGACGGCCCCTGCTTCAATGGTGGACAGTGTGCTGACAACCCAG  1610
short 1         ----------------------------------------------------------------------
short 2         ----------------------------------------------------------------------

dld wt    1161  AGGGAGGATATTTCTGCCAGTGCCCGATGGGTTATGCTGGATTCAACTGTGAGAAGAAGATCGATCACTG  1680
short 1         ----------------------------------------------------------------------
short 2         ----------------------------------------------------------------------

dld wt    1681  CAGCTCCAACCCTTGCTCGAATGATGCTCAGTGTCTCGATCTTGTGGACTCCTATCTTTGCCAGTGTCCT  1750
short 1         ----------------------------------------------------------------------
short 2         ----------------------------------------------------------------------

dld wt    1751  GAGGGATTCACAGGAACGCACTGCGAAGACAACATCGACGAGTGTGCCACCTATCCCTGCCAGAATGGCG  1820
short 1         ----------------------------------------------------------------------
short 2         ----------------------------------------------------------------------

dld wt    1821  GCACTTGCCAAGACGGACTCAGCGACTACACCTGCACCTGCCCGCCTGGATACACCGGCAAGAACTGCAC  1890
short 1         ----------------------------------------------------------------------
short 2         ----------------------------------------------------------------------

dld wt    1891  CTCTGCGGTCAACAAGTGCCTCCACAACCCTTGCCACAACGGTGCCACTTGTCATGAAATGGACGGTCGA  1960
short 1         ----------------------------------------------------------------------
short 2         ----------------------------------------------------------------------

dld wt    1961  TATGTGTGCGCTTGCATCCCAGGTTATGGAGGACGCAACTGTCAGTTCTTACTCCCTGAAAACCCACAAG  2030
short 1         ----------------------------------------------------------------------
short 2         ----------------------------------------------------------------------

dld wt    2031  GACAAGCCATCGTTGAGGGAGCCGACAAGAGATACTCTTACGAAGAAGACGACGGTGGTTTTCCATGGAC  2100
short 1         ----------------------------------------------------------------------
short 2         ----------------------------------------------------------------------

dld wt    2101  GGCGGTTTGCGCTGGGATTATTTTAGTGCTTTTAGTGCTGATCGGCGGCTCCGTCTTTGTCATTTACATC  2170
short 1         ----------------------------------------------------------------------
short 2         ----------------------------------------------------------------------

dld wt    2171  CGTCTCAAGCTGCAGCAGAGGAGCCAGCAAATCGATAGCCATAGTGAAATCGAGACCATGAACAACCTGA  2240
short 1         ----------------------------------------------------------------------
short 2         ----------------------------------------------------------------------

dld wt    2241  CCAACAACCGCAGCCGAGAGAAGGACTTGTCCGTAAGCATCATCGGAGCCACGCAAGTGAAAAACATCAA  2310
short 1         ----------------------------------------------------------------------
short 2         ----------------------------------------------------------------------

dld wt    2311  CAAGAAAGTGGACTTTCAGAGCGACGGCGACAAAAACGGATTCAAATCGCGATACTCGCTAGTGGATTAC  2380
short 1         ----------------------------------------------------------------------
short 2         ----------------------------------------------------------------------

dld wt    2381  AATCTTGTTCATGAGCTGAAGCAGGAGGACTTGGGGAAAGAGGATTCTGAGAGGAGCGAAGCCACAAAAT  2450
short 1         ----------------------------------------------------------------------
short 2         ----------------------------------------------------------------------
```

```
dld wt   2451  GCGAGCCTCTCGATTCCGACTCAGAGGAGAAGCACAGAAATCATTTAAAAAG------------------  2502
short 1        ----------------------------------------------------------------------
short 2        --------------------------GAGAAGCACAGAAATCATTTAAAAAGgtaaatgagtgtgaattt

dld wt   2503  --------------------------------------------------------------TGACTCCT  2510
short 1        ----------------------------------------------------------------------
short 2        tggctagcgcagatccatttgtcgatgtaagcagtactaaagttgcctcttttcgttttcagTGACTCCT

dld wt   2511  CAGAGAGGAAACGAACAGAATCTTTGTGCAAGGACACAAAGTACCAGTCAGTCTTTGTTTTATCAGAGGA  2580
short 1        ----------------------------------------------------------------------
short 2        CAGAGAGGAAACGAACAGAATCTTTGTGCAAGGACACAAAGTACCAGTCAGTCTTTGTTTTATCAGAGGA

dld wt   2581  GAAAGATGAATGTATTATTGCAACCGAGGTGTAACGCCACAACAGAGCTGTAGCTCATCTTGTTCTACAG  2650
short 1        -----------------------------------------ACAGAGCTGTAGCTCATCTTGTTCTACAG
short 2        GAAAGATGAATGTATTATTGCAACCGAGGTGTAACGCCACAACAGAGCTGTAGCTCATCTTGTTCTACAG

dld wt   2651  GAGGTTTTACTCCAGTAATGCTGCTCAAACCAGAGGGAAGGTTCCCTTCACGTGACTGCTGCTGTGAAAC  2720
short 1        GAGGTTTTACTCCAGTAATGCTGCTCAAACCAGAGGGAAGGTTCCCTTCACGTGACTGCTGCTGTGAAAC
short 2        GAGGTTTTACTCCAGTAATGCTGCTCAAACCAGAGGGAAGGTTCCCTTCACGTGACTGCTGCTGTGAAAC

dld wt   2721  TAATGTTGAAGGTGATATTCAGAATGAGCTGGTTCTCAACTGAAGTAAACACAGTGACTGCAGGAAAGGA  2790
short 1        TAATGTTGAAGGTGATATTCAGAATGAGCTGGTTCTCAACTGAAGTAAACACAGTGACTGCAGGAAAGGA
short 2        TAATGTTGAAGGTGATATTCAGAATGAGCTGGTTCTCAACTGAAGTAAACACAGTGACTGCAGGAAAGGA

dld wt   2791  CGTTGGGCCATACTGGCTCGGACTCTCAAAGTTTGTTTCCCAGGAACGTGCCAAATGGACTTGCTTGCAC  2860
short 1        CGTTGGGCCATACTGGCTCGG
short 2        CGTTGGGCCATACTGGCTCGG

dld wt   2861  TTCCTGTAGGGACGTTTTTTTTTTACCTCATTGCACTATGATGGGTTGTATTTTTATTGTATTTAACACA  2930
dld wt   2931  CAATTGAATCGAATACTGTGTATGCATATGCACTGACTGCAGTGTGCATCTGGAAGTTCTCGGATCCTTG  3000
dld wt   3001  GATCGCGAGAGGAAGAACACTGCCATCAATGATGTTTGTAACTGATTCGTTGTTTTATTTTCGGAAAGCT  3070
dld wt   3071  TGTGTTTTCTTTATTTAAATGTTATTTGTGATCGACGTGTTTGTGGTTTACTTTGTGTGTGTGTGTGTGT  3140
dld wt   3141  GAAGTTTGATATAATTTAAAATCTTTTTTTCATTTCGCAGATATGTCTATTGTAAATAAGGCACTTCGGG  3210
dld wt   3211  TCGACGTGACTGTTCTCTGTAAATGTATTTAAACGTGCTGGTAAAACTTACAGGAGTTGTTTTTATGACT  3280
dld wt   3281  GTTGTCCAATGAAAAAAAAATCACCTTAATATTTTTCCAAATAAATCCTGTATGAAACTTCAAAAAAAAA  3350
dld wt   3351  AAAAAAAAAAAAAAAAAAAAAAAAAAA                                              3377
```
