# Supplementary material for: Rbm24a and Rbm24b Are Required for Normal Somitogenesis
Source: PLoS One. 2014 Aug 29;9(8):e105460. doi: 10.1371/journal.pone.0105460 (PMC4149414; doi:10.1371/journal.pone.0105460)
Supplement: Table S1 — Primer sequences. Primers used to generate antisense DIG labeled riboprobes, conduct splice variant RT-PCR and conduct qRT-PCR are listed. (PDF) [file pone.0105460.s008.pdf]

Riboprobe	Forward 5' - 3'	Reverse 5' - 3'
rbm24a	CCAGGGGTTATGGATTTGTG	TGCAGTTGTTGGGGTTGATA
rbm24b	CGGAGGTCTTCCCTATCACA	CCAAACGCACACAAGAGCTA
myf5	CAATCACGCCTTTGAGGCAC	GGGAATCACTTCCGGTTGGA
myod	ACCCTTGCTTCAACACCAAC	GCCCATAAAATCCATCATGC
myog	GGATCGAAGAAAGGCCGCTA	GCCTCTGTTCCCGTTATGC
myf6	ACCTGTTTGAGACCAACGCT	TCTGAAGACTCCAACACGGC
fgf8a	AGCTTTACAGCCGAACCAGT	AGTAGCGGGTGCGTTTAGTC
tbx16	TCACCAAACCTGGCAGAAGG	AGAGCTTCACACGATGACGG
ntla	AGACGAATGTTTCCCGTGCT	GGTCTGGGACTTCCTTGTGG
dlc	GTGCACCTACGGCACCGGAA	CACTTGCACTCACCGGGGGC
dld	ATTCCTTTCGCGTTTGGGTTCACATGGCCA	TCCGTCTTGGCAAGTGCCGCC
notch1a	ACCCGTGTCTGAACCAGGGCT	TCCCGGCAAACACACGCAGG
notch3	AGCACCAAGGTATTCAGCAGC	TGGCGGCTCTTCAATAACAGC
her7	CATATCCTCATTGATATCAAC	AGAGATTACACAAGGCCCATCA
dla	TGGAAACCCCTGCCGCAACG	GACTACTGCGCACGCCACGA
dlb	ATCTCCGCAGAGCCCCCGTG	AAAGTCGGTGGGCATCGGCA
dll4	CAACTTTGGGTGGCCGGGGT	CTGGCCAGTGAAGCCCGCTC

Splice PCR	Forward 5' - 3'	Reverse 5' - 3'
dlc	TCGGACTACTCTCACAGTCTGCT	TGTGGTCAGGCCCACTGGTGT
dld	TGCCTGGCCCGTGGAAGTTT	CCGAGCCAGTATGGCCCAACG
her1	TCGTCTTCTTCCGATTTTCAGCC	ACAACGCTGGTTATTCTCATGCT
her7	CGATGAAAGACCTCCACCTGC	GGGGGAGAAGAAATCTGCGT
fgf8a	TGCATGTACTTGTCAGCGGT	TTGGACCCCATAGACTTTCATTGT
gli2a	TTCGTGACAGAGTCCAAGCC	GTGCACTGCTGAGAAAGGGA
pax3a	TCAAGTGCTTCGTGCATCCT	TCTGACATTATTGAGAGCAATTCAA
smo	CCCGAGAACGGAGGCATAAA	CAGAATGAGCACCAGTAACTCCA
tbx6	TGAAGAAAGAGCAGACCGAGA	GGTCCTCCGATGCAGTGTTT

RT-PCR	Forward 5' - 3'	Reverse 5' - 3'
elfalpha	TCTGTTACCTGGCAAAGGGG	GGAGTCGACGTGGCCAATAA
dlc	TCGTGTCGTGTGCGATGAAT	ACAGCCAGACAAGCAGATGG
dld	GTCTTCCGGGGTGTGATGAA	GGCACGGTTTGTGATGTGTG
myod	CTGCCCAAAGTGGAGATTCTG	TTCTCGTCTGACACGTTGGG
dld short 1	GAGCATTACTACGGCGAGGG	AGCTACAGCTCTGTTGTGCAG
dld short 2	TGGCTAGCGCAGATCCATTT	TGGCGTTACACCTCGGTTG
